# The activation mechanism of Irga6, an interferon-inducible GTPase contributing to mouse resistance against *Toxoplasma gondii*

**DOI:** 10.1186/1741-7007-9-7

**Published:** 2011-01-28

**Authors:** Nikolaus Pawlowski, Aliaksandr Khaminets, Julia P Hunn, Natasa Papic, Andreas Schmidt, Revathy C Uthaiah, Rita Lange, Gabriela Vopper, Sascha Martens, Eva Wolf, Jonathan C Howard

**Affiliations:** 1 Institute for Genetics, Department of Cell Genetics, University of Cologne, Zülpicher Strasse 47a, 50674 Cologne, Germany; 2 Max-Planck-Institute for Molecular Physiology, Department of Structural Biology, Otto-Hahn-Strasse 11, 44227 Dortmund, Germany; 3 Current address: Institute of Biochemistry II, Medical Faculty of the Goethe University, University Hospital Building 75, Theodor-Stern_Kai 7, 60528 Frankfurt am Main, Germany; 4 Current address: Crucell Holland BV, Archimedesweg 6, 2333 CN Leiden, Netherlands; 5 Current address: National University of Singapore, Division of Bioengineering, Block E3A, #07-02 7, Engineering Drive 1, 117576 Singapore; 6 Current address: The Rockefeller University, 1230 York Avenue, New York, NY 10065, USA; 7 Current address: Max F. Perutz Laboratories, University of Vienna, Dr. Bohrgasse 9/3, 1030 Vienna, Austria; 8 Current address: Max-Planck-Institute of Biochemistry, Department of Structural Cell Biology, Am Klopferspitz 18, 82152 Martinsried, Germany

## Abstract

**Background:**

The interferon-inducible immunity-related GTPases (IRG proteins/p47 GTPases) are a distinctive family of GTPases that function as powerful cell-autonomous resistance factors. The IRG protein, Irga6 (IIGP1), participates in the disruption of the vacuolar membrane surrounding the intracellular parasite, *Toxoplasma gondii*, through which it communicates with its cellular hosts. Some aspects of the protein's behaviour have suggested a dynamin-like molecular mode of action, in that the energy released by GTP hydrolysis is transduced into mechanical work that results in deformation and ultimately rupture of the vacuolar membrane.

**Results:**

Irga6 forms GTP-dependent oligomers *in vitro *and thereby activates hydrolysis of the GTP substrate. In this study we define the catalytic G-domain interface by mutagenesis and present a structural model, of how GTP hydrolysis is activated in Irga6 complexes, based on the substrate-twinning reaction mechanism of the signal recognition particle (SRP) and its receptor (SRα). In conformity with this model, we show that the bound nucleotide is part of the catalytic interface and that the 3'hydroxyl of the GTP ribose bound to each subunit is essential for *trans*-activation of hydrolysis of the GTP bound to the other subunit. We show that both positive and negative regulatory interactions between IRG proteins occur via the catalytic interface. Furthermore, mutations that disrupt the catalytic interface also prevent Irga6 from accumulating on the parasitophorous vacuole membrane of *T. gondii*, showing that GTP-dependent Irga6 activation is an essential component of the resistance mechanism.

**Conclusions:**

The catalytic interface of Irga6 defined in the present experiments can probably be used as a paradigm for the nucleotide-dependent interactions of all members of the large family of IRG GTPases, both activating and regulatory. Understanding the activation mechanism of Irga6 will help to explain the mechanism by which IRG proteins exercise their resistance function. We find no support from sequence or G-domain structure for the idea that IRG proteins and the SRP GTPases have a common phylogenetic origin. It therefore seems probable, if surprising, that the substrate-assisted catalytic mechanism has been independently evolved in the two protein families.

## Background

Immunity-related GTPases (IRG proteins/p47 GTPases) are major contributors to cell autonomous resistance against the intracellular protozoal pathogen, *Toxoplasma gondii *[1-3]. For nomenclature of IRG proteins, see Methods and [4]. Multiple members of the family are expressed in cells induced by interferon-γ (IFNγ). Many IRG proteins, including Irga6 (IIGP1) and Irgb6 (TGTP) relocate from resting cytoplasmic compartments to the parasitophorous vacuole membrane (PVM) of avirulent *T. gondii *[1,5,6]. Loading of IRG proteins onto the *T. gondii *PVM is followed by vesiculation and rupture of the PVM and death of the parasite [5,7,8]. Irga6 at the PVM is in the active, GTP-bound state, while cytoplasmic Irga6 is inactive and probably GDP-bound [9,10].

Irga6 forms GTP-dependent oligomeric complexes *in vitro *and *in vivo *and hydrolysis of the GTP substrate is cooperatively activated [10,11]. These enzymatic properties of Irga6 together with the relatively high molecular mass of 47 kDa and the nucleotide binding affinities in the micromolar range [11] are also found in several other families of large GTPases, including members of the dynamin superfamily [12], associated with membrane remodelling and, like Mx proteins, resistance against intracellular pathogens [1,13].

The structure of the Irga6 protein was determined some years ago [14]. The protein consists of a Ras-like G-domain [15] and a helical domain (Additional file 1). The G-domain contains three conserved GTP-binding motifs (G1, G3 and G4) [16] and two flexible switch regions, switch I and switch II [17]. Homology considerations suggest that the structure of Irga6 can provide a reasonable template for the IRG family. Three members of the IRG family, Irgm1 (LRG-47), Irgm2 (GTPI) and Irgm3 (IGTP), carry a unique substitution of the otherwise universally conserved P-loop (G1 motif) lysine (GKS subfamily) to methionine (GMS subfamily) (Additional file 2) [4,18]. In the absence of GDP-dependent negative regulatory interactions with the three GMS proteins, GKS subfamily members including Irga6 activate prematurely in the cytoplasm, form GTP-dependent aggregates, and are unable to accumulate on the PVM of invading *T. gondii *[9,10,19].

Little is known about the relationship between the GTP-dependent activation of Irga6 and pathogen resistance. Our study poses some specific questions directed towards an understanding of these processes at a molecular level: where are the interfaces that participate in oligomerisation and interactions with other IRG proteins, how is GTP hydrolysis activated in the oligomeric complexes, and finally, is oligomeric complex formation required for resistance against *T. gondii*? We carried out an extensive mutagenesis screen to address the first question and found a novel interface of Irga6 located in the G-domain. This interface is required for oligomerisation and for accelerated hydrolysis of GTP. From experimental analysis of this interface we can propose a structural model for the activation of GTP hydrolysis that is, surprisingly, based on the hydrolytic mechanism of the signal recognition particle (SRP) and its receptor (SRα) [20,21]. We demonstrate that the catalytic interface includes the bound GTP substrate and that the 3'hydroxyl (3'OH) of the nucleotide ribose is required for activation of hydrolysis in *trans*. We also show the engagement of the catalytic interface in both the activating interaction of Irga6 with Irgb6 [6,9] and the inhibitory interaction between Irga6 and the GMS subfamily protein, Irgm3 [9]. Lastly, we show that the integrity of the catalytic interface of Irga6 is required for the accumulation of the active, GTP-bound protein at the *T. gondii *PVM.

## Results

### The catalytic interface is localised on the G-domain

**Figure 1:**
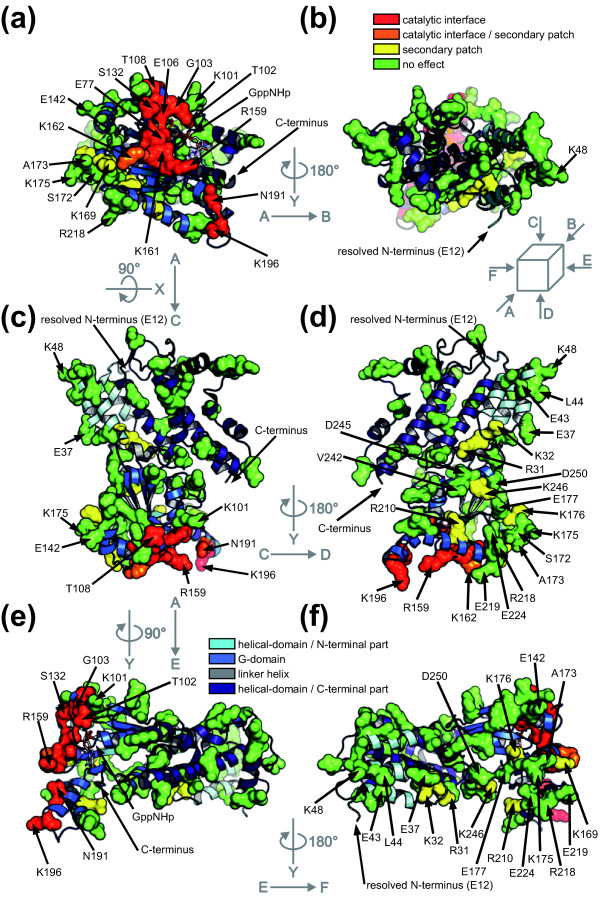
**Position of mutated residues**. Mutated residues are shown in the structure of Irga6-M173A [14]. The protein backbone is shown as follows: the N-terminal helical domain (cyan), the G-domain (light-blue), the linker-helix (gray) and the C-terminal helical domain (dark blue). The surface formed by the following residues is shown: (i) Glu77, Thr102, Gly103, Glu106, Thr108, Ser132, Arg159, Lys161, Asp164, Asn191 and Lys196 define the catalytic interface (red); (ii) Lys162 is located at the border of the catalytic interface and the secondary patch (orange); (iii) Arg31, Lys32, Lys169, Lys176, Arg210 and Lys246 define the secondary patch (yellow); (iv) Ser18, Glu37, Glu43, Leu44, Lys48, Asn50, Gln52, Ser56, Glu64, Thr88, Glu97, Lys101, Met109, Glu110, Arg111, Lys115, Glu142, Lys145, Glu148, Asp150, Ser172, Ala173 (instead of Met173), Lys175, Glu177, Lys202, Glu203, Arg218, Glu219, Glu224, His237, Val242, Asp245, Asp250, Lys255, Asn265, Ser269, Arg275, Glu285, Asn293, Ser304, Lys310, Lys311, Thr325, Ser326, Glu335, Lys346, Asp355, Glu356, Glu357, Leu372, Ala373 and Lys407 did not prevent oligomerisation, when mutated (green). Lys9 and Ser10 are not resolved in the crystal structure. **(a) **Front view of the G-domain. **(b) **Rear view; Figure 1a rotated by 180° around y-axis. **(c) **Top view; Figure 1a rotated by 90° around x-axis. **(d) **Bottom view; Figure 1c rotated by 180° around y-axis. **(e) **Right view; Figure 1a rotated by 90° around y-axis. **(f) **Left view; Figure 1e rotated by 180° around y-axis.

**Figure 2:**
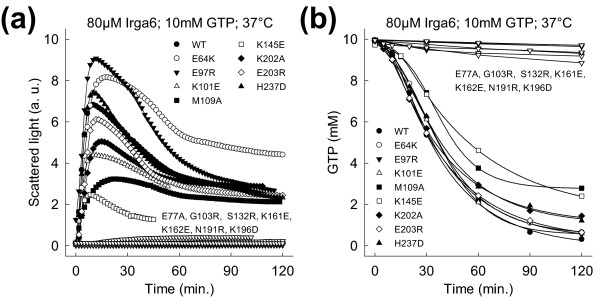
**The G-domain is involved in Irga6 oligomerisation**. **(a) **Oligomerisation of 80 μM WT or mutant Irga6 proteins was monitored by light scattering in the presence of 10 mM GTP at 37°C. **(b) **Hydrolysis of 10 mM GTP (with traces α^32^P-GTP) was measured in the presence of 80 μM WT or mutant Irga6 proteins at 37°C. Samples were assayed by TLC and autoradiography.

To gain insight into the mechanism of GTP-dependent oligomerisation and activation of Irga6, a number of solvent-exposed residues identified from the known crystal structure [14] were mutated (Figure 1), in large part with bulky charged residues with the intention of disrupting putative intermolecular interactions. Mutations of a contiguous cluster of G-domain residues (Glu77, Gly103, Glu106, Ser132, Arg159, Lys161, Lys162, Asp164, Asn191, Lys196) essentially abolished oligomerisation (Figure 2a and see below) and GTP hydrolysis (Figure 2b and see below). Mutations of two further residues in this cluster, Thr102 and Thr108, have been shown elsewhere to lose both functions [22]. These residues define a surface (Figure 1a, red and orange), called the catalytic interface, that includes the conformationally labile switch I region (residues 100 to 109), suggesting that the conformation of switch I is critical for dimer formation and activation.

Mutations of the residues Arg31, Lys32, Lys169, Lys176, Arg210 and Lys246 (Figure 1, yellow) also reduced GTP-dependent oligomerisation to some extent (Additional file 3), but none completely (compare Additional file 4 and 5). These residues formed a loosely defined "secondary patch" on the Irga6 surface (Figure 1d, f). Unlike the catalytic interface, however, the secondary patch is interspersed with residues which, when mutated, had no effect on oligomerisation, and indeed a substantial part of the secondary patch area could be replaced simultaneously without preventing oligomerisation (data not shown). At present, therefore, we do not consider the secondary patch to be an oligomerisation interface. The oligomerisation of Irga6 was not prevented by numerous other mutations (Figure 1, green), suggesting the absence of a second well-defined surface interface contributing to oligomerisation.

The majority of catalytic interface mutants including T102A and T108A [22] had no significant effect on the binding affinity for GTP (Additional file 6). Thus the failure of these mutants to oligomerise is not caused by reduced nucleotide binding. The mutations E77A, R159E, K161E and N191R slightly decreased the nucleotide binding affinity (Additional file 6) but it is unlikely that this caused the loss of oligomerisation because the G4-motif mutant, Irga6-D186N, with a considerably lower binding affinity for guanine nucleotides, oligomerised relatively efficiently in the presence of GTP (see below). Furthermore, none of the mutants of the secondary patch, which all showed reduced nucleotide-binding affinities (Additional file 6), prevented oligomerisation of Irga6.

Irga6 crystallizes as a rotationally symmetrical dimer [14] (Additional file 7). Mutants of the crystal dimer interface were seen to oligomerise less efficiently than the wild-type (WT) and it was suggested that this interface might participate in cooperative GTP-dependent activation [14]. The crystal dimer interface does not obstruct the catalytic interface described here (Additional file 8) and could therefore contribute to active Irga6 oligomerisation. Mutants of the four crystal dimer interface residues Leu44, Lys48, Ser172 and Met173, that had been examined earlier [14], were therefore re-assayed (Additional file 9). Mutants of nine further residues (Glu37, Glu43, Glu142, Lys169, Lys175, Lys176, Glu177, Arg218 and Glu224) in the crystal dimer interface were also analysed (Additional file 3). Under the conditions of these experiments, which were more stringent than those used previously, out of 13 residues mutated in the crystal dimer interface only the mutations of Lys169 and Lys176, two residues already identified in the secondary patch, partially inhibited oligomerisation (Additional file 7). Furthermore, the formation of the crystal dimer is not nucleotide-dependent [14], whereas the oligomerisation of Irga6 requires GTP binding [11]. These arguments urge that the crystal dimer interface does not identify the oligomerisation interface associated with activation. Thus no convincing second interface required for oligomerisation has yet been found on the surface of the known crystal structure [14] of Irga6 (Figure 1), suggesting that oligomerisation requires a cryptic interface exposed following GTP binding or dimer formation at the catalytic interface.

### The catalytic interaction of the SRP GTPases provides a scaffold for a model of the Irga6 dimer

**Figure 3:**
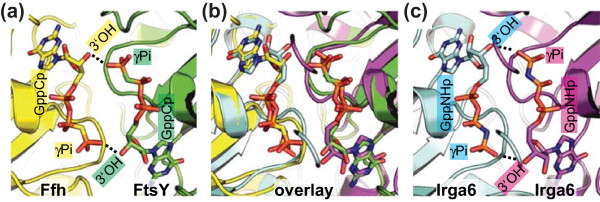
**Construction of the Irga6 dimer model**. Views of the nucleotide-binding regions involved in formation of the dimers. **(a) **Crystal structure of the Ffh (yellow) FtsY (green) heterodimer (PDB 1RJ9) [20]. **(b) **Two molecules (cyan and magenta) of Irga6-M173A (PDB 1TQ6) [14] were adjusted to the Ffh-FtsY heterodimer, to give the best overlay for the bound nucleotides. **(c) **The model of the Irga6 dimer is shown. The *trans *interactions of the 3'OHs with the γ-phosphates are represented as dotted lines.

Although usually discussed in the context of dynamin and its relatives, Irga6 also resembles in several respects two GTPases of the SRP family, Ffh (SRP54 homologue) and FtsY (SRα homologue). Ffh, FtsY and Irga6 share a wide-open nucleotide-binding pocket in their nucleotide-bound state [14,23,24]. Ffh [25], FtsY [26] and Irga6 [11] share nearly identical low nucleotide-binding affinities, caused by a high substrate dissociation rate. Ffh and FtsY are significantly homologous to each other and form a GTP-dependent heterodimer. Each acts in *trans *as a GTPase activating protein (GAP) for the other in the dimeric complex [27]. Irga6 molecules work as mutual GAPs in GTP-dependent oligomeric complexes [11]. The common biochemical and structural properties suggested that the mechanism of cooperative hydrolysis known from Ffh and FtsY could be relevant for understanding the catalytic mechanism of Irga6. The key to this idea is the catalytic interaction between the two closely opposed GTP molecules in anti-parallel orientation and, specifically, the crystallographically determined, catalytically important reciprocal *trans *interactions between the 3'OHs and the γ-phosphates [20,21]. Coordinates of the nucleotides from the crystal structure of the Ffh-FtsY heterodimer from Egea and colleagues (PDB 1RJ9) [20] (Figure 3a) were used to define the position in space of the nucleotides bound by two Irga6 molecules (Figure 3b and 3c). The Irga6-M173A GppNHp complex structure (Additional file 1) was used for this analysis since this structure was the only one that resolved all residues between Glu12 and the C-terminal Asn413 (PDB 1TQ6) [14].

**Figure 4:**
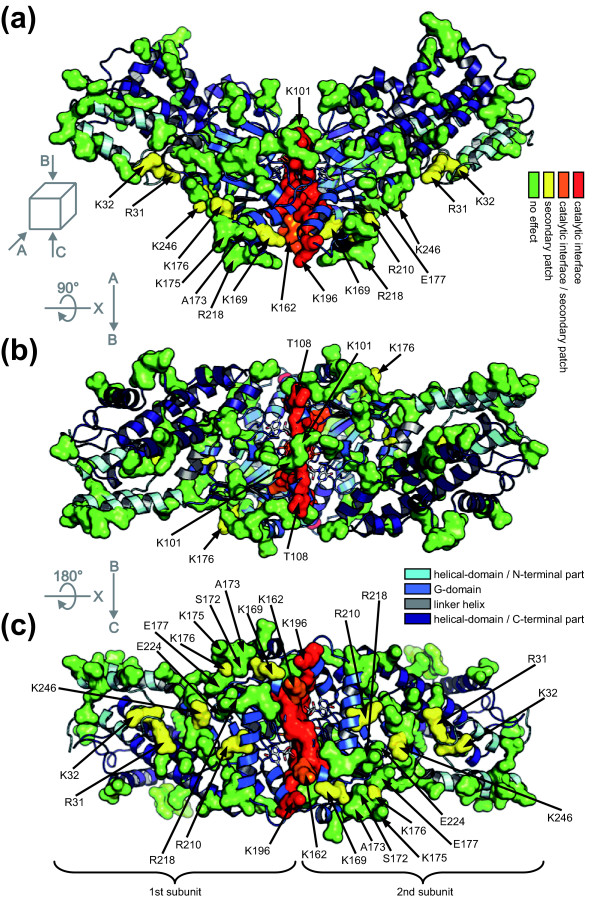
**Position of mutated residues in the Irga6 dimer model**. Model of the Irga6 dimer including the catalytic interface (Figure 3c) is shown. Protein domains and mutated residues are shown as indicated in the Figure 1. **(a) **Front view. **(b) **Top view; Figure 4a rotated by 90° around x-axis. **(c) **Bottom view; Figure 4b rotated by 180° around x-axis.

In the Irga6 dimer model thus constructed (Figure 4 and Additional file 10) the two Irga6 molecules complement each other well, apart from one overlap of the two atomic structures made by the side chain of Arg159 (Additional files 11, 12 and 13). The buried surface area in the hypothetical Irga6 dimer is 2400 Å^2 ^(Additional file 8a, b). The modeled catalytic interface surface is in good agreement with the mutagenesis data. All the residues where mutation destroyed oligomerisation are located within or proximal to the contact area of the dimer subunits (Figure 4). The only exception is Lys101, localised just inside the margin of the catalytic interface surface though oriented outwards (Figure 4a, b), where mutation had no effect on oligomerisation (Figure 2a). The properties of Lys101 and Arg159 may indicate the occurrence of conformational changes that, by analogy with Ffh-FtsY [20,21], are expected to accompany GTP binding and complex formation.

### The ribose of the bound nucleotide is part of the catalytic interface

**Figure 5:**
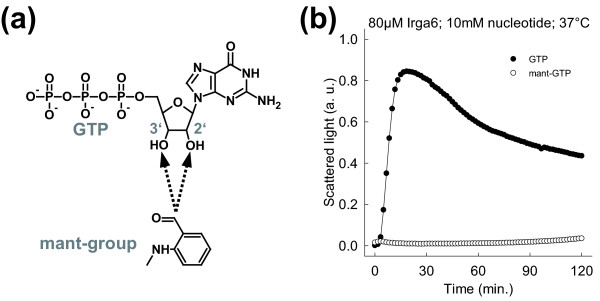
**The nucleotide ribose is part of the catalytic interface**. **(a) **Mant-GTP; the mant-group is attached via the 2' and the 3'oxygen to the GTP ribose respectively. The attachment places are indicated by dotted arrows. **(b) **Oligomerisation of 80 μM WT Irga6 protein was monitored by light scattering in the presence of 10 mM GTP or mant-GTP at 37°C.

At the core of the Irga6 dimer model the 2' and 3'OHs of GTP ribose form part of the contact surface. Modifications of the nucleotide ribose at the 2' or 3'OH would therefore be expected to interfere with oligomerisation. Oligomerisation of Irga6 in the presence of 2'/3'O-(N-methylanthraniloyl)-GTP (mant-GTP) was investigated. Mant is a small fluorescent group bound via the 2' or 3'oxygen to the GTP ribose in mant-GTP, a nucleotide analog used in affinity determinations (Figure 5a). Consistent with the lack of free space between the subunits of the dimer model, mant-GTP was unable to stimulate oligomerisation of Irga6 (Figure 5b). Mant-group-dependent inhibition of complex formation was also observed for Irga6 protein immunoprecipitated from cells (N. Papic, unpublished data). The inhibitory effect of the mant-group is not caused by reduced nucleotide binding [11], implying as predicted from the dimer model that the GTP ribose is part of the interaction interface between Irga6 oligomer subunits.

### The base of bound nucleotide is part of the catalytic interface

**Figure 6:**
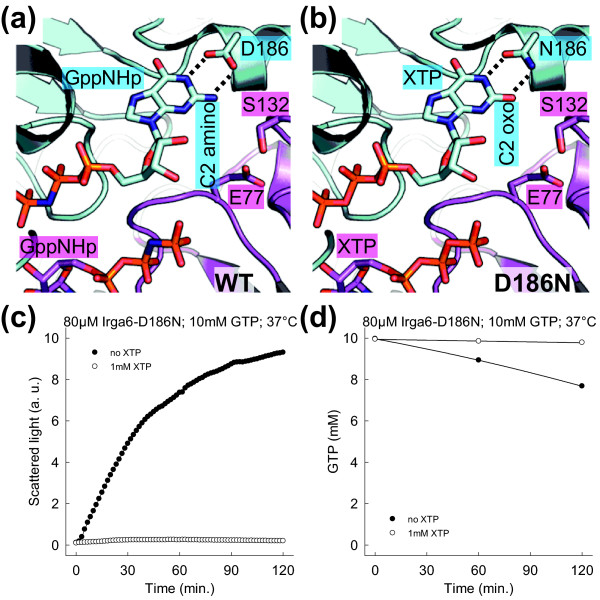
**The nucleotide base is part of the catalytic interface**. **(a and b) **View of the nucleotide-binding region. The Irga6 dimer model (Figure 4) is shown. Glu77, Ser132 (magenta), Asp186 (cyan), of WT Irga6, with two GppNHp nucleotides **(a) **and modeled Asn186 (cyan), of Irga6-D186N, with two XTP nucleotides **(b) **are shown. The interactions of Asp186 with GppNHp and of Asn186 with XTP are represented by dotted lines. **(c) **Oligomerisation of 80 μM Irga6-D186N protein was monitored by light scattering in the presence of 10 mM GTP at 37°C. The experiment was performed with and without the addition of 1 mM XTP. **(d) **Hydrolysis of 10 mM GTP (with traces α^32^P-GTP) was measured in the presence of 80 μM Irga6-D186N protein at 37°C. The experiment was performed with and without the addition of 1 mM XTP. Samples were assayed by TLC and autoradiography.

In the Irga6 dimer model the bound nucleotides are part of the interaction interface of the two subunits. The specificity of GTPases for guanine nucleotides is determined by a conserved aspartate in the G4-motif. This aspartate interacts with the exocyclic amino-group of the guanine ring at the C2 position (Figure 6a). The substitution of the G4 aspartate by asparagine changes the nucleotide specificity of GTPases from guanine to xanthine nucleotides, which have an oxo-group at the C2 position (Figure 6b). The D251N mutation in the G4-motif of Ffh changes the binding preference of the protein from GTP to xanthosine-5'triphosphate (XTP). It was shown that GTP-initiated complex formation between the two SRP GTPases, Ffh-D251N and FtsY, is inhibited by addition of XTP [28]. The C2 amino-group is part of the interaction surface between Ffh and FtsY therefore the binding of XTP to Ffh-D251N alters the interface and inhibits complex formation.

The nucleotide-binding preference of Irga6 was changed from guanine to xanthine based nucleotides by the corresponding G4-motif mutation D186N (Additional file 14). Unexpectedly, despite a nine-fold higher affinity for XTP than for GTP, Irga6-D186N hydrolysed GTP more efficiently than XTP (Additional file 15). Oligomerisation of Irga6-D186N (Figure 6c) accompanied by GTPase activity (Figure 6d) could be activated by GTP, albeit inefficiently, and both were abolished when the high affinity ligand, XTP, was added at a concentration 1/10 that of GTP (Figure 6c, d). This shows that the replacement of the surface exposed C2 amino-group, of bound GTP, by the oxo-group, of XTP, inhibits Irga6 oligomerisation, implicating the nucleotide base as part of the interaction interface between the complex-forming molecules, as in Ffh-FtsY. In the dimer model the two relatively close *trans *neighbours of the GTP base C2 amino-group are Glu77 and Ser132 (Figure 6a). Consistently, mutations of Glu77 and Ser132 both caused loss of oligomerisation (Figure 2a).

### The 3'OH of the GTP ribose is required for *trans*-activation of GTP hydrolysis

**Figure 7:**
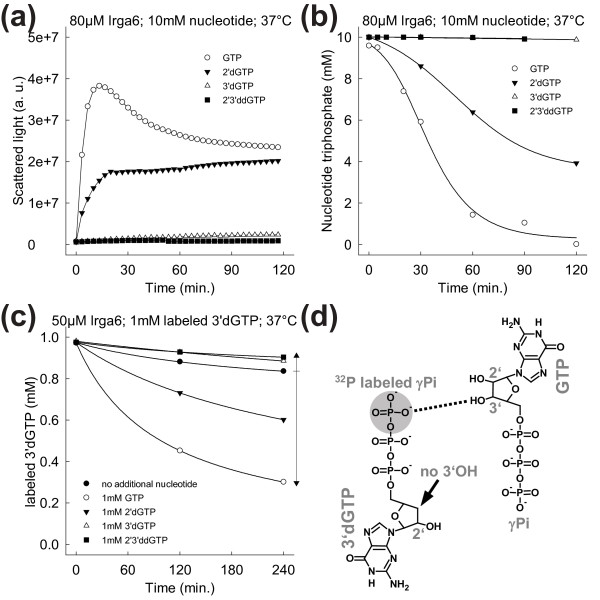
**The GTP ribose 3'OH is essential for the activation of GTP hydrolysis in *trans***. **(a) **Oligomerisation of 80 μM WT Irga6 protein was monitored by light scattering in the presence of 10 mM GTP, 2'dGTP, 3'dGTP or 2'3'ddGTP at 37°C. **(b) **Hydrolysis of 10 mM GTP, 2'dGTP, 3'dGTP or 2'3'ddGTP was measured in the presence of 80 μM WT Irga6 protein at 37°C. Samples were assayed by HPLC. **(c) **Hydrolysis of 1 mM 3'dGTP (with traces γ^32^P-3'dGTP) was measured in the presence of 50 μM WT Irga6 protein at 37°C. The experiment was performed with and without the addition of 1 mM unlabeled GTP, 2'dGTP, 3'dGTP or 2'3'ddGTP. Samples were assayed by TLC and autoradiography. **(d) **Model of the interaction between labeled 3'dGTP and unlabeled GTP in the core of the Irga6 dimer. The radioactively labeled γ-phosphate of the 3'dGTP is marked with a gray circle. The putative activatory *trans *interaction between the 3'OH of GTP and the γ-phosphate of 3'dGTP is represented as a dotted line.

The model of the Irga6 dimer is based on reciprocal *trans *interactions between the ribose 3'OHs and the γ-phosphates of the opposed nucleotides [20,21], analogous to those shown to be crucial for the reciprocal activation of GTP hydrolysis between the paired GTPases, Ffh and FtsY [20,27]. The involvement of the 2' and 3'OHs of GTP in Irga6 oligomerisation and GTP hydrolysis was investigated. Oligomerisation of Irga6 could be stimulated by GTP and 2'deoxy-GTP (2'dGTP), both of which have the 3'OH. In contrast, no complex formation was observed in presence of 3'deoxy-GTP (3'dGTP) and 2'3'dideoxy-GTP (2'3'ddGTP), both of which lack the 3'OH (Figure 7a and Additional file 16a). Consistent with these results, only basal hydrolysis rates of about 0.02 min^-1 ^were found for 3'dGTP and 2'3'ddGTP in contrast to efficient hydrolysis of GTP or 2'dGTP (Figure 7b). The oligomerisation rate in presence of 2'dGTP was somewhat reduced (Figure 7a), consistent with the idea that the 2'OH is part of the catalytic interface, as suggested by the model. But, unlike the 3'OH the 2'OH is not required for cooperative hydrolysis (Additional file 16b). The removal of the 2' or 3'OH of the GTP ribose decreased the nucleotide-binding affinity slightly (Additional file 14). However, as already argued, the K_d _variation alone cannot be responsible for the observed inability of 3'dGTP and 2'3'ddGTP to stimulate Irga6 oligomerisation at millimolar nucleotide concentrations.

For the Ffh-FtsY heterodimer the essential activation function of the 3'OH is mediated in *trans *[20]. We, therefore, investigated whether the basal hydrolysis of radioactively labeled 3'dGTP could be enhanced by addition of unlabeled GTP, 2'dGTP, 3'dGTP or 2'3'ddGTP. Since each Irga6 monomer has only one nucleotide-binding site, an increase in 3'dGTP hydrolysis by addition of GTP must be due to an activation by a second, GTP-loaded, monomer in *trans*. Furthermore, *trans*-activation of hydrolysis of 3'dGTP, a nucleotide which itself does not contain the 3'OH, would show the dispensability of the 3'OH in *cis*. Consistent with the Irga6 dimer model the addition of GTP and 2'dGTP stimulated the hydrolysis of labeled 3'dGTP, whereas the addition of 3'dGTP and 2'3'ddGTP had an inhibitory effect (Figure 7c). Therefore, the 3'OH is required in *trans *but not in *cis *for the activation of hydrolysis. A model of the dimer interaction responsible for the *trans *activation of hydrolysis of labeled 3'dGTP by unlabeled GTP is shown in Figure 7d.

### Glu106 is a key residue crucial for the activation of catalysis

**Figure 8:**
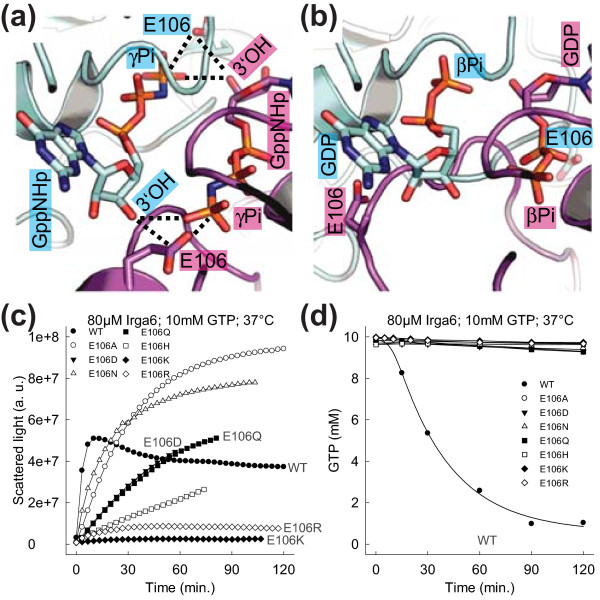
**The Glu106 is essential for the activation of GTP hydrolysis**. **(a and b) **View of the nucleotide-binding region. **(a) **The Irga6 dimer model (Figure 4) is shown (cyan and magenta). The *cis *interaction between the Glu106 and the γ-phosphate, and the putative *trans *interactions between the 3'OH and Glu106, as well as between the 3'OH and the γ-phosphate are represented by dotted lines. **(b) **Two molecules (cyan and magenta) of Irga6 bound to GDP (PDB 1TPZ/A) [14] were adjusted to the Irga6 dimer model, to give the best overlay for the G1, G3, G4 and G5-motifs. The resulting theoretical model of the "Irga6 dimer in the GDP state" is shown. **(c) **Oligomerisation of 80 μM WT or mutant Irga6 proteins was monitored by light scattering in the presence of 10 mM GTP at 37°C. **(d) **Hydrolysis of 10 mM GTP (with traces α^32^P-GTP) was measured in the presence of 80 μM WT or mutant Irga6 proteins at 37°C. Samples were assayed by TLC and autoradiography.

The core of the Irga6 dimer model is created by the reciprocal *trans *interactions of the GTP 3'OHs with the γ-phosphates. These enforce a specific relative orientation of the two nucleotides and, therefore, also of the two protein molecules to which the nucleotides are bound. The dimer model suggests additional *trans *interactions between the 3'OHs and the Glu106 residues (Figure 8a). The involvement of Glu106 in the activation of GTP hydrolysis was investigated. Glu106 is part of the catalytic interface (Figure 1a). The mutants E106K and E106R essentially abolished oligomerisation (Additional file 17). However some exchanges like E106D, E106Q, E106A and E106N, could oligomerise to a considerable extent (Figure 8c). Nevertheless, no mutation of Glu106 could activate hydrolysis, whether oligomerising or not (Figure 8d). Thus, Glu106 is a residue essential for the activation of GTP hydrolysis independently of a role in the contact interface.

Glu106 is part of the flexible switch I region which undergoes nucleotide-dependent conformational changes [14]. In the GDP state Glu106 is exposed and points away from the bound nucleotide, a spatial arrangement that is incompatible with the formation of the dimer as suggested by the model (Figure 8b). However, in the GppNHp state Glu106 can be reoriented towards the γ-phosphate of the bound nucleotide [14]. The GTP ribose 3'OH may stabilize the Glu106 residue in *trans *in a conformation allowing complex formation and in an orientation required for activation of the catalytic water molecule in *cis *(Figure 8a). This could initiate a nucleophilic attack on the γ-phosphate and activate GTP hydrolysis.

### The catalytic interface of Irga6 is essential for heteromeric interactions between IRG members

**Figure 9:**
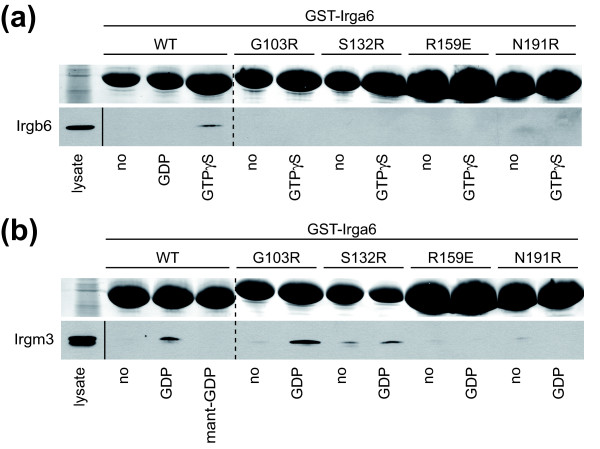
**The catalytic interface is involved in Irga6-Irgb6 and Irga6-Irgm3 interactions**. Pull-down of Irgb6 **(a) **and Irgm3 **(b) **with recombinant GST-tagged Irga6 protein from IFNγ-stimulated gs3T3 fibroblasts lysate in the presence or absence of guanine nucleotides (0.5 mM GDP, GTPγS or mant-GDP). GST-Irga6 protein was visualised by Ponceau S staining upon blotting (top rows). Irgb6 and Irgm3 were detected with anti-Irgb6 and anti-Irgm3 monoclonal antibodies (bottom rows). A shorter exposure of the lysate input is shown. Dotted lines indicate positions where irrelevant lanes were removed from the image.

In addition to forming GTP-dependent homomeric complexes *in vitro *[11] and *in vivo *[10] Irga6 can also form heteromeric nucleotide-dependent complexes with other members of the IRG family. The GKS protein, Irgb6, interacts strongly with Irga6 in yeast 2-hybrid assays in a nucleotide-dependent manner [9]. This is a positive interaction that assists the accumulation of Irga6 on the PVM of *T. gondii *[6,9]. We could observe this interaction in a pull-down assay between recombinant glutathione S-transferase (GST)-tagged Irga6 and Irgb6 from IFNγ-induced cell lysates (Figure 9a). The interaction was GTPγS-dependent and failed in the presence of GDP (Figure 9a). The importance of the catalytic interface for this interaction was demonstrated by the complete failure of GTPγS-dependent pull-downs with catalytic interface mutants of Irga6 (Figure 9a).

The three GMS proteins, Irgm1, Irgm2 and Irgm3, are essential negative regulators of Irga6 [9]. For Irgm3 this interaction has been shown to be GDP-dependent and inhibited by GTPγS [9]. In cells, in the absence of this interaction, Irga6 binds GTP, activates spontaneously, and cannot accumulate on PVMs of invading *T. gondii *[9,10]. We were able to confirm the previously documented GDP-dependent interaction between IFNγ-induced Irgm3 and recombinant Irga6 in a pull-down assay, and additionally showed that no interaction occurred when mant-GDP was used (Figure 9b), hinting at usage of the catalytic interface. This was confirmed when two of four mutants of the catalytic interface also blocked the GDP-dependent interaction of Irgm3 with Irga6 (Figure 9b). The two residues whose mutation did not interfere with Irga6-Irgm3 interaction, Gly103 and Ser132, are located in a different part of the catalytic interface from Arg159 and Asn191. These results suggest that the GDP-dependent negative regulatory interaction between Irgm3 and Irga6 indeed involves the catalytic interface, but with a slightly different orientation or a higher affinity from that of the GTP-dependent activating interaction.

### The catalytic interface is required for recruitment of Irga6 to the *T. gondii *PVM

**Figure 10:**
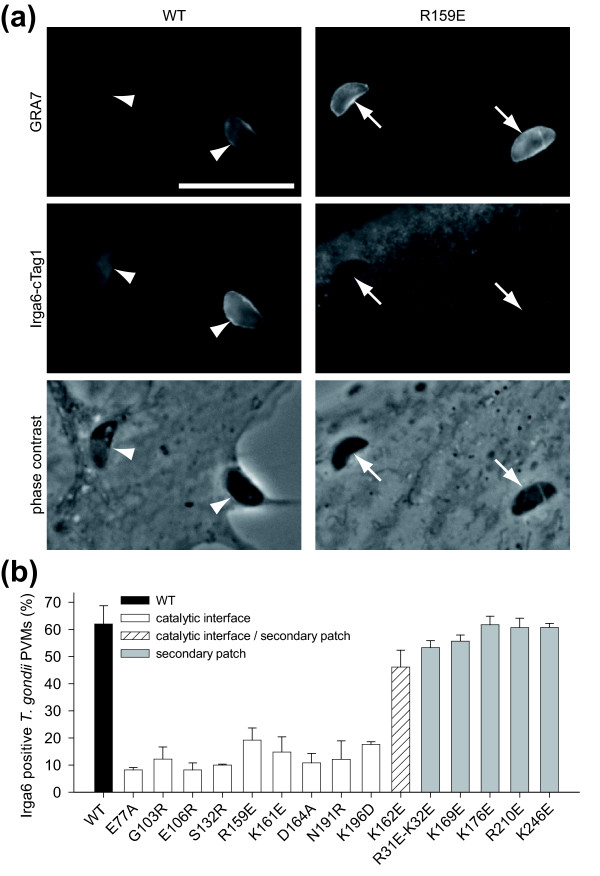
**The catalytic interface is required for efficient targeting of Irga6 to the *T. gondii *PVM**. Irga6-deficient MEFs were stimulated with IFNγ and transiently transfected with an Irga6-cTag1 construct. The cells were infected with the avirulent *T. gondii *strain ME49. Intracellular parasites were detected with anti-GRA7 monoclonal antibody and ectopically expressed Irga6 with anti-cTag1 antiserum. **(a) **Representative images of the WT and a mutant of the catalytic interface are shown. Irga6 coated (arrowhead) and non-coated (arrow) parasites are indicated. Scale bar, 10 μm. **(b) **Irga6 positive PVMs were counted among the total amount of intracellular parasites. The mean values of two independent experiments are shown. The error bars indicate the standard deviation between individual experiments.

In IFNγ stimulated cells about 60% of Irga6 colocalises with the endoplasmic reticulum (ER) while the remainder is cytosolic [19]. Upon infection with *T. gondii *the protein accumulates on the PVM [5,6], depleting other cytoplasmic compartments. IRG protein-dependent destruction of the PVM and subsequent death of the parasite occurs in IFNγ-induced cells [8,19]. It is not known whether the process of Irga6 oligomer formation is causally connected with immunity against *T. gondii*. This issue was addressed in the context of the accumulation of Irga6 at the *T. gondii *PVM [5]. Irga6-deficient cells, stimulated with IFNγ and transiently transfected with WT or mutant Irga6, were infected with the avirulent *T. gondii *strain ME49 (Figure 10a). WT Irga6 accumulated on the PVM, while all mutants of the catalytic interface showed quantitatively (Figure 10b) and qualitatively (Additional file 18) drastically reduced recruitment to the PVM. The sole exception was Lys162, located at the rim of the catalytic interface and the secondary patch (Figure 1a). Mutations of this residue inhibited oligomerisation and catalytic activity *in vitro *(Figure 2) but did not prevent accumulation of the mutant protein on the PVM (Figure 10b). The contribution of Lys162 to the catalytic interface is ambiguous: the structural model places the residue just inside the interface. It is possible that the configuration of the Irga6 catalytic interface *in vivo *differs slightly from that *in vitro*. This could be due to a conformational effect of the myristoyl group, which is exposed by GTP binding *in vivo *[10] and required for vacuolar accumulation (N. Papic, unpublished data). Equally, the predominant catalytic dimers that form *in vivo *may be heterodimers between Irga6 and other IRG proteins rather than Irga6 homodimers [6,9]. Heterodimeric catalytic interfaces may be slightly different from the Irga6 homodimer interface assayed here.

## Discussion

Irga6 forms GTP-dependent oligomers and GTP hydrolysis is activated in this state [11]. The present study has identified a new catalytic interface (Figure 1) required for the formation of Irga6 oligomers. This interface provides a platform for both positive and negative nucleotide-dependent regulatory interactions between Irga6 molecules and other members of the IRG protein family (Figure 9). These interactions are essential for the activity of the IRG proteins in resistance to *T. gondii *(Figure 10) [9]. The revealed surface is part of the G-domain, including the nucleotide-binding site and the switch regions (Figure 1). The nucleotide itself is part of the interface (Figures 5 and 6). Structural and biochemical features common to the SRP GTPases and Irga6 suggested a model (Figure 3) for the Irga6 dimer based on the relative orientation of the two nucleotides buried in the SRP-SRα complex [20,21]. The mutagenesis data were consistent with the proposed model (Figure 4), but the key to the activation of GTP hydrolysis by SRP and SRα in the dimeric complex is the reciprocal *trans *interaction between the 3'OH of the GTP ribose and the γ-phosphate of the two nucleotides [20,21]. In strong support for the validity of the SRP-SRα based model of the Irga6 dimer, the 3'OH of the GTP ribose proved to be absolutely required for oligomerisation and GTP hydrolysis by Irga6 and, as in SRP-SRα, this function was exercised in *trans *only (Figure 7).

Functionally, Irga6 seems closer to the dynamins in that it is involved in the vesiculation and disruption of the PVM [5], yet the catalytic geometry appears far closer to the SRP GTPases. Despite this distinctive similarity, however, the IRG and SRP protein families appear to be completely unrelated to each other in sequence in those parts of the molecule that compose the catalytic interface (Additional file 19) and belong, in fact, to the two different major clades (SIMIBI and TRAFAC) that have been defined over multiple GTPase families [29]. If indeed, IRG proteins share the unusual catalytic mechanism of SRP [20,21] then these proteins appear to represent convergent approaches to the same solution. For the SRP GTPases it is clear that the solution is ancient, but until a convincing ancestry for the IRG proteins is found it is not possible to say whether their organization is ancient or derived.

### Alternative Irga6 dimer models

So far, the majority of dimeric GTPases for which structure is known engage the two monomers in a parallel orientation and the two nucleotides are separated and do not interact [1,30,31]. In contrast, the SRP-SRα paired GTPases engage the two monomers in an anti-parallel orientation with the two nucleotides in reciprocal atomic contact [20,21]. We explored the feasibility of alternative models of the Irga6 dimer based on the relative orientation of the nucleotides, and consequently of the G-domains, found in the dimeric structures of other GTPases and related ATPases (data not shown). None provided a satisfactory basis on which to explain the properties of Irga6. In models based on EHD2 [32], MeaB [33] and MnmE [34], the two Irga6 G-domains interact via different surfaces that do not include the bound nucleotides. The models based on SEPT2 [35], GIMAP2 [36], BDLP [37], Toc34 [38] and Soj [39] engage small parts of the catalytic interface in limited interfaces. The catalytic interface is involved in the models based on HypB [40] and Av2 [41]. The model based on hGBP1 [42] involves the catalytic interface but the subunits overlap in the contact area. The dynamin [43] based model would involve the catalytic interface, if the subunits were closer. However, none of the alternative models engaging the catalytic interface bring the two nucleotides into atomic contact. None of the models of the Irga6 dimer except that based on SRP-SRα offer an explanation for the critical requirement in *trans *of the 3'hydroxyl of the GTP ribose for the activation of catalysis (Figure 7c).

### The activation of GTP hydrolysis in Irga6 complexes

GAPs work by supplementation of missing catalytic residues (arginine finger; asparagine thumb), and by reorientation and stabilization of the catalytic machinery which is already present in the target protein [44-46]. The model of the Irga6 dimer suggests that the switch regions are stabilized by the interaction of the two Irga6 molecules. In particular, the model suggests that Glu106 (switch I) is stabilized by the *trans *interaction with the 3'OH of the GTP ribose (Figure 8a). Mutational analysis of Glu106 (Figure 8c, d) together with structural data [14] urge that this residue activates the catalytic water molecule for the nucleophilic attack on the γ-phosphate in *cis *and is therefore crucial for the activation of GTP hydrolysis. The finding that the 3'OH of the GTP ribose is essential for activation of GTP hydrolysis in *trans *(Figure 7c) is consistent with the anticipated function of Glu106.

On complex formation between Ffh and FtsY catalytic residues of the switch I region become reoriented and facilitate GTP hydrolysis in *cis*. It is proposed that aspartates activate the catalytic water molecules, and that arginines coordinate the γ-phosphates [20,21,47]. In contrast to the Irga6 dimer model, there is no *trans *interaction between the 3'OHs and the catalytic aspartates in the Ffh-FtsY complex; the catalytic aspartates approach the γ-phosphates from a different direction [20,21]. It may be relevant that acidic residues have been implicated in activating the catalytic water in further dimer-forming GTPases, MeaB [33], MnmE [34] and HypB [40], as also in related ATPases, Soj [39] and Av2 [41].

The Irga6 dimer model does not suggest any positively-charged residue that could fulfill an arginine finger-like function. The mutation of the most promising candidate, Lys101 in switch I, to glutamate had no effect on complex formation or GTP hydrolysis (Figure 2). The non-necessity of an arginine finger-like residue was demonstrated for Ran and Rap; instead, in both cases a tyrosine OH was found to interact with the γ-phosphate, and, in the case of Ran, also with the catalytic glutamine [48,49]. These interactions recall the proposed *trans *interactions of the 3'OH of the GTP ribose with the γ-phosphate and Glu106 in the Irga6 dimer model (Figure 8a). Generally, the transition state in Irga6 could be stabilized in *cis *and in *trans *by hydrogen bond donation from the residues surrounding the nucleotide and also from water molecules that, by analogy to Ffh-FtsY [20,21], potentially bridge the two opposed nucleotides.

### The catalytic interface - a general interaction platform involved in activation and regulation

The catalytic interface is the most conserved part of the Irga6 surface (Additional file 20). It probably represents a central platform engaged in functional interactions between IRG proteins in general. Heteromeric interactions between Irga6 and other members of the IRG family play important regulatory roles in the biological action of Irga6. While Irgb6 enhances the accumulation of activated Irga6 [10] on the *T. gondii *PVM [6,9], Irgm3 prevents the premature activation of Irga6 prior to infection by locking the GDP-bound state of the protein [9]. *In vitro*, the catalytic interface is involved in the GTP-dependent Irga6-Irgb6 interaction (Figure 9a) and also in the GDP-dependent Irga6-Irgm3 interaction (Figure 9b). These results show that the negative regulatory interaction between Irga6 and Irgm3 occurs, like the activating Irga6-Irga6 and Irga6-Irgb6 interactions, via the catalytic interface. The outcome of the Irga6-Irgm3 interaction thus resembles the primary action of a GDP dissociation inhibitor (GDI) [9]. Thus two different functions (GAP and GDI) seem to be mediated through the catalytic interface. All tested mutants of the catalytic interface prevented the Irga6-Irgb6 interaction (Figure 9a), but the Irga6-Irgm3 interaction was prevented only by a subset of the mutants (Figure 9b) suggesting a distinct mode of interaction. The catalytic interface of the GMS proteins, including Irgm3, contains specific substitutions (Additional file 2). The otherwise conserved residues Glu106, Asp164 and Arg159, which are crucial for oligomerisation and GTP hydrolysis, are substituted by arginine, histidine and glutamine respectively in the GMS proteins. The corresponding mutations, E106R, D164H and R159Q in Irga6 have deleterious effects on GTP-dependent complex formation and hydrolysis activation (Figure 8 and Additional file 11). The specific modifications of the catalytic interface in GMS proteins may facilitate complex formation with GDP-bound GKS proteins, thus prolonging their inactive state in the absence of infection.

### The catalytic interface plays a central role in the antimicrobial function and is a target for a *T. gondii *virulence factor

Upon infection with avirulent *T. gondii *Irga6 accumulates at the PVM and participates in disruption of the PVM and killing of the parasite [5,8]. The accumulation of IRG proteins at the PVM is a prerequisite for the antimicrobial function [5,6,8]. The biological importance of complexes formed via the catalytic interface is shown by the fact that mutations of this surface strongly diminish the accumulation of Irga6 on the PVM of avirulent *T. gondii *(Figure 10). Irga6, with other IRG proteins, does not accumulate normally on the PVM of virulent *T. gondii *and the parasites survive and continue to replicate [6,8,50]. The secreted ROP18 kinase [51] is a major virulence factor of *T. gondii *[52]. The significance of the catalytic interface for the function of Irga6 is highlighted by the recent finding that the virulence-associated ROP18 kinase from virulent, but not avirulent, *T. gondii *strains phosphorylates conserved threonine residues, Thr102 and Thr108, in switch I within the catalytic interface of Irga6, thus blocking oligomerisation, GTPase activity and the accumulation of Irga6 at the PVM [22].

## Conclusions

An intracellular way of life can protect pathogens from antibodies, but hosts can deploy other specialized defense mechanisms against such pathogens. *Toxoplasma gondii*, is an intracellular protozoal pathogen of mammals and birds, and commonly infects humans. Mice exploit a specialized intracellular resistance system, the immunity-related GTPases (IRG proteins), for defense against *T. gondii*. The IRG protein, Irga6, accumulates rapidly on the membrane surrounding intracellular parasites. Shortly after, this membrane ruptures and the parasite dies. The enzymatic activity, required for the antimicrobial function, of Irga6 is activated in oligomeric complexes formed by the protein.

We define one of the contact surfaces involved in Irga6 oligomerisation, the so-called catalytic interface, which is a part of the G-domain and to which the bound nucleotide contributes. This strongly conserved interface participates in the positive and negative regulatory interactions of Irga6 with Irgb6 and Irgm3 respectively, thus it is a universal platform engaged in interactions between and regulation of IRG proteins. The catalytic interface is essential for the accumulation of Irga6 on the membrane, surrounding *T. gondii *within infected cells, and is therefore required for the antimicrobial function of the protein.

Further, we propose a model for the dimer formed via the catalytic interface of Irga6, based on the unique substrate geometry and catalytic machinery found in the dimeric complex of the signal recognition particle, Ffh (SRP54), and its receptor, FtsY (SRα). The reciprocal catalytic interaction, made in *trans *by the 3'hydroxyl of the bound nucleotide ribose, determines the relative orientation of the signal recognition particle and its receptor in the dimeric complex. The 3'hydroxyl of the nucleotide ribose is also essential for Irga6 complex formation and activation of GTP hydrolysis in *trans*. The model also explains how a catalytic glutamate residue is engaged in the activation of catalysis.

Since there is no distinctive sequence homology between the SRP GTPases and Irga6, we consider that the functional similarity between these two GTPase families is probably the result of convergent evolution.

## Methods

### Nomenclature of IRG proteins

Irga6, the main subject of this study was originally named IIGP [18,53]. The name was later modified to IIGP1 and biochemical [11] and structural [14] studies on the protein were performed under this name. The nomenclature of the whole protein family was rationalized in 2005 under the generic name IRG (immunity-related GTPases) to accommodate its genomic structure and phylogenetic complexity [4], and IIGP1 was renamed Irga6.

### Expression constructs and mutagenesis

Expression constructs were generated by site directed mutagenesis in pGEX-4T-2-Irga6 [11] and pGW1H-Irga6-cTag1 [10] using the QuickChange protocol (Stratagene, La Jolla, CA, USA). Primers used (including reverse complement sequences) are listed in Additional file 21.

### Recombinant protein expression and purification

Irga6 protein was expressed as N-terminal GST fusions from pGEX-4T-2 constructs in *Escherichia coli *BL21 upon overnight induction with 0.1 mM IPTG at 18°C. The cells were lysed in PBS (137 mM NaCl, 2.7 mM KCl, 10.1 mM Na_2_HPO_4_, 1.8 mM KH_2_PO_4_, pH 7.4)/2 mM DTT/Complete Mini Protease Inhibitor Cocktail EDTA free (Roche, Grenzach-Wyhlen, Germany) using a microfluidiser (EmulsiFlex-C5; Avestin, Ottawa, Ontario, Canada). Cleared lysates were purified on a GSTrap FF glutathione Sepharose affinity column (GE Healthcare, Munich, Germany) in PBS/2 mM DTT. GST was cleaved off by overnight incubation of the resin with thrombin (Serva, Heidelberg, Germany) at 4°C. Irga6 was eluted with PBS/2 mM DTT. Protein containing fractions were subjected to size exclusion chromatography (Superdex 75; GE Healthcare, Munich, Germany) in B1 buffer (50 mM Tris/HCl pH 7.4, 5 mM MgCl_2_)/2 mM DTT. Irga6 containing fractions were concentrated with Vivaspin 20 centrifugal concentrator (Sartorius, Goettingen, Germany). When indicated, an abbreviated protein purification procedure was used; omitting size exclusion chromatography and purifying Irga6 by glutathione affinity chromatography only.

### Oligomerisation assays

Oligomerisation of Irga6 was monitored in B1 buffer/2 mM DTT by conventional or dynamic light scattering (DLS). For both, conventional and DLS, the protein buffer solution (90 μl) was cleared by ultracentrifugation (100,000 g, 30 minutes, 4°C). The reaction was started by addition of ice cold nucleotide (10 μl) to the protein buffer solution. The reaction was mixed by pipetting and transferred immediately to a cuvette. Conventional light scattering was performed at 350 or 600 nm at 37°C in an Aminco-Bowman 2 Luminescence Spectrometer (SLM Instruments, Urbana, IL, USA) or a DM45 Spectrofluorimeter (Olis, Bogart, GA, USA). Due to the unit-less readout the values obtained from the two instruments cannot be directly compared. DLS was performed at 650 nm at 20°C or 37°C with a DynaPro-E-20-660 molecular sizing instrument (Protein Solutions; Wyatt Technologies, Santa Barbara, CA, USA). Data were obtained and analysed using the DYNAMICS 5 and 6 software. Values of hydrodynamic radius given on the ordinates reflect the population mean particle size. Note that the derived hydrodynamic radius is not equal to the real size of the oligomer. WT and mutant Irga6 (with the exception of D164R and D164K) were stable and did not aggregate at 37°C in the presence of GDP.

### Nucleotide hydrolysis assays

Nucleotide hydrolysis was measured in B1 buffer/2 mM DTT either by thin layer chromatography (TLC) and autoradiography or by high performance liquid chromatography (HPLC). For TLC and autoradiography, Irga6 was incubated with the indicated amounts of unlabeled nucleotide and traces of radioactively labeled nucleotide. The reaction was separated on PEI Cellulose F TLC plates (Merck, Darmstadt, Germany) in 1 M acetic acid/0.8 M LiCl. Signals were detected with the BAS 1000 phosphoimager analysis system (Fujifilm, Duesseldorf, Germany) and quantified with AIDA Image Analyser 3 (Raytest, Straubenhardt, Germany) or ImageQuant TL 7 (GE Healthcare, Munich, Germany) software. For HPLC, the reaction was stopped by 10-fold dilution in 10 mM NaOH; nucleotides were separated by ion exchange chromatography (MiniQ 4.6/50 PE; GE Healthcare, Munich, Germany) in 10 mM NaOH over a NaCl gradient. Absorption at 254 nm was monitored. Unicorn 4.12 (GE Healthcare, Munich, Germany) was used for quantification of peak areas.

### Nucleotide binding measurement

Nucleotide-binding affinities were determined by equilibrium titration of Irga6 in the range of 0 to 100 μM against 0.5 mM mant nucleotide in B1 buffer/2 mM DTT at 20°C. The mant nucleotide was excited at 355 nm, and monitored at 448 nm in an Aminco-Bowman 2 Luminescence Spectrometer (SLM Instruments, Urbana, IL, USA). Equilibrium dissociation constants were obtained as described by [54]. SigmaPlot 9 (Systat, Chicago, IL, USA) was used for dissociation constant (K_d_) calculation.

### Pull-down

IFNγ-induced (200 U/ml) gs3T3 cells were lysed for one hour at 4°C in PBS/0.1% Thesit (Sigma-Aldrich, St.Louis, MO, USA)/3 mM MgCl_2 _/Complete Mini Protease Inhibitor Cocktail EDTA free (Roche, Grenzach-Wyhlen, Germany). Postnuclear supernatants were incubated at 4°C overnight with glutathione Sepharose 4B (GE Healthcare, Munich, Germany)-bound recombinant GST-Irga6 with 0.5 mM of the indicated nucleotide. Cellular proteins were eluted from the washed beads with 100 mM Tris/HCl pH 8.5/0.5% SDS for 30 minutes at room temperature and subjected to SDS-PAGE and Western blot. Irgb6 and Irgm3 were detected using mouse monoclonal antibodies B34 [55] and anti-IGTP (BD Biosciences, Franklin Lakes, NJ, USA) respectively. Input of recombinant GST-Irga6 was monitored by Ponceau S staining.

### Cell culture and *T. gondii *infection

*T. gondii *ME49 tachyzoites were passaged and used for infection of Irga6-deficient mouse embryonic fibroblasts (MEFs) [5] as described earlier [6]. MEFs were transiently transfected with pGW1H-Irga6-cTag1 [10] constructs using FuGENE 6 (Roche, Grenzach-Wyhlen, Germany) and stimulated with 200 U/ml IFNγ (Peprotech, Hamburg, Germany) for 24 hours followed by infection with *T. gondii *at a multiplicity of infection of 7 for 2 hours, synchronised by centrifugation. Cells were fixed in 3% paraformaldehyde for 15 minutes and used for indirect immunostaining.

### Immunocytochemistry

Immunocytochemistry was performed as described earlier [5] using anti-cTag1 rabbit sera [5], anti-GRA7 mouse monoclonal antibodies [56,57] and Alexa 488/555 labeled donkey anti-rabbit and anti-mouse sera (Molecular Probes, Darmstadt, Germany). Probes were analysed microscopically as described earlier [6]. Intracellular parasites were identified by the staining pattern of the *T. gondii *protein GRA7.

### Nucleotides

GTP (Carl Roth, Karlsruhe, Germany and Sigma-Aldrich, St.Louis, MO, USA); GDP (Sigma-Aldrich, St.Louis, MO, USA); GTPγS, XTP, 2'deoxy-GTP, mant-GTP, mant-GDP, mant-GTPγS, 2'mant-3'deoxy-GTP, 2'deoxy-3'mant-GTP, mant-XTP and mant-XDP (Jena Bioscience, Jena, Germany); 3'deoxy-GTP (Jena Bioscience, Jena, Germany and Trilink Biotechnologies, San Diego, CA, USA); 2'3'dideoxy-GTP (GE Healthcare, Munich, Germany); α^32^P-GTP (GE Healthcare, Munich, Germany, Hartmann Analytic, Braunschweig, Germany and Perkin Elmer, Waltham, MA, USA); γ^32^P-3'dGTP (Hartmann Analytic, Braunschweig, Germany)

### Software

Swiss-PdbViewer [58] was used for construction of structural models. CNSsolve [59] module buried surface [60] was used for calculation of contact surfaces. ClustalW2 [61] was used for protein sequence alignment generation. ConSurf [62,63] was used for calculation of conservation. PyMOL 0.99 (DeLano Scientific, Palo Alto, CA, USA) was used for image generation.

## Abbreviations

DLS: dynamic light scattering; ER: endoplasmic reticulum; GAP: GTPase activating protein; GDI: GDP dissociation inhibitor; GST: glutathione S-transferase; HPLC: high performance liquid chromatography; IFN: interferon; IRG: immunity-related GTPase; mant: methylanthraniloyl; MEF: mouse embryonic fibroblast; OH: hydroxyl; PVM: parasitophorous vacuole membrane; SR: signal recognition particle receptor; SRP: signal recognition particle; TLC: thin layer chromatography; WT: wild-type; XTP: xanthosine-5'triphosphate.

## Competing interests

The authors declare that they have no competing interests.

## Authors' contributions

All authors performed aspects of the research. The manuscript was prepared by NP, EW and JCH.

## Supplementary Material

Additional file 1 **The three-dimensional structure of Irga6**. The Crystal structure of Irga6-M173A GppNHp (PDB 1TQ6) [14] is shown. Protein domains are shown as indicated in the Figure 1. **(a to f) **The same orientations of the molecule are shown as in Figure 1. Click here for file

Additional file 2 **Amino acid sequence alignment of mouse IRGs**. Amino acid sequence alignment of Irga1, Irga2, Irga3, Irga4, Irga6, Irga7, Irga8, Irgb1, Irgb2, Irgb3, Irgb4, Irgb5, Irgb6, Irgb8, Irgb9, Irgb10, Irgd, Irgm1, Irgm2 and Irgm3 from the C57BL/6 mouse. Irgc is not induced by IFNγ, Irga5 and Irgb7 are pseudogenes [4] and were thus excluded. Residues relevant for the crystal dimer interface (CDI) (Additional file 8c and 8d) are highlighted (yellow 1 - red 6; indication how often they form part of the crystal dimer interface in the three available dimeric structures of Irga6 [14]). Residues that are part of the catalytic interface (CI) (Additional file 8a, b) are marked (black X). Residues mutagenised (MUT) in this study and by Steinfeldt *et al*. [22] (Figure 1) are indicated: no inhibition of oligomerisation (green 0), inhibition of oligomerisation and part of the secondary patch (yellow 1), inhibition of oligomerisation and part of the secondary patch or the catalytic interface (orange 2), inhibition of oligomerisation and part of the catalytic interface (red 3). The calculated conservation score (CON) (Additional file 20) is displayed: variable (cyan 1) - conserved (magenta 9). The G1, G3, G4 and G5-motifs are highlighted by a red box. The GKS and GMS subfamilies are separated by a green line. Click here for file

Additional file 3 **Mutants of the secondary patch reduce oligomerisation**. Mutagenesis of surface residues. **(a) **Oligomerisation of partially purified (see Methods) 80 μM WT or mutant Irga6 proteins was monitored by light scattering in the presence of 10 mM GTP at 37°C. Left panel: positive (WT) and negative (K196D) control (Figure 2a). Right panel: investigated mutants. Five mutants R31E-K32E, K169E, K176E, R210E and K246E inhibited the oligomerisation of Irga6, whereas many others had no significant effect. The mutants were fully purified. **(b) **Oligomerisation of 80 μM WT or mutant Irga6 proteins was monitored by light scattering in the presence of 10 mM GTP at 37°C. **(c) **Hydrolysis of 10 mM GTP (with traces α^32^P-GTP) was measured in the presence of 80 μM WT or mutant Irga6 proteins at 37°C. Samples were assayed by TLC and autoradiography. Click here for file

Additional file 4 **Oligomerisation of the catalytic interface mutants**. Oligomerisation of 80 μM Irga6 mutant proteins was monitored in the presence of 10 mM GDP or GTP by DLS at 37°C. Click here for file

Additional file 5 **Oligomerisation of the secondary patch mutants**. Oligomerisation of 80 μM Irga6 mutant proteins was monitored in the presence of 10 mM GDP or GTP by DLS at 37°C. Click here for file

Additional file 6 **Nucleotide-binding affinities of oligomerisation impaired Irga6 mutants**. Dissociation constant (K_d_) measured by equilibrium titration. The mean values and the standard deviation of at least two independent experiments are shown. Click here for file

Additional file 7 **Position of mutated residues in the crystal dimer**. The Irga6 crystal dimer (PDB 1TPZ) [14] is shown. Protein domains and mutated residues are shown as indicated in the Figure 1. Lys9, Ser10, Lys196 of both subunits and Lys202 of the second subunit are not resolved in the crystal structure. **(a) **Top view. **(b) **Front view of the two G-domains; Additional file 7a rotated by 90° around the x-axis. **(c) **Left view; Additional file 7b rotated by 90° around the y-axis. Click here for file

Additional file 8 **Relative position of catalytic and crystal dimer interface**. The structure of Irga6-M173A [14] is shown. Protein domains are shown as indicated in the Figure 1. **(a and b) **Residues buried in the interface of the Irga6 dimer model were calculated with CNSsolve [59] module buried surface [60] with a probe radius of 1.4 Å. The surface formed by Glu77, Thr78, Gly79, Asn94, Glu95, Lys101, Thr102, Gly103, Glu106, Val107, Gly131, Ser132, Thr133, Pro136, Pro137, Ala157, Thr158, Arg159, Phe160, Lys161, Lys162, Asn163, Asp166, Lys184, Asp186, Ser187, Asp188, Thr190, Asn191, Asp194, Gly195 and Lys233 is shown in magenta. **(c and d) **Residues buried in the crystal dimer interface were calculated by the same method. The two surfaces formed by Asn14, Ser18, Gln36, Glu37, Asn40, Leu41, Glu43, Leu44, Arg47, Lys48, Pro137, Asn138, Thr139, Leu141, Glu142, Tyr147, Asp166, Ala168, Lys169, Ala170, Ser172, Ala173 (instead of Met173), Met174, Lys175, Lys176, Glu177, Phe178, Arg218, Gly221, Ile222, Ala223 and Glu224 are shown. Three dimeric crystal structures of Irga6 are available (PDB 1TPZ, 1TQ2 and 1TQD) [14] therefore each residue can be maximum six time involved in this interface. Residues highly relevant for the crystal dimer interface are shown in red, less relevant in yellow. **(a and c) **Front view of the G-domain (Figure 1a). **(b and d) **Left view (Figure 1f). Click here for file

Additional file 9 **Mutations of the crystal dimer interface do not prevent oligomerisation**. **(a) **Oligomerisation of 80 μM WT or mutant Irga6 proteins was monitored by light scattering in the presence of 10 mM GTP at 37°C. **(b) **Hydrolysis of 10 mM GTP (with traces α^32^P-GTP) was measured in the presence of 80 μM WT or mutant Irga6 proteins at 37°C. Samples were assayed by TLC and autoradiography. Click here for file

Additional file 10 **Irga6 dimer model**. Atomic coordinates (structural PDB file) of the constructed (Figure 3) Irga6 dimer model (Figure 4). Click here for file

Additional file 11 **Asp164 and Arg159 participate in oligomerisation**. For the construction of the Irga6 dimer model a rigid crystal structure was used. In the model the side chains of the Arg159 residues of the two subunits collide. Arg159 is located close to Asp164 on the other subunit. Asp164 forms the bottom of a pocket, derived from two loops. One loop is located between Glu77 and Ser80 and contains a part of the G1-motif. The other loop is located between Ile155 and Asn163. The conformation of Arg159 is relatively unconstrained [14]. A conformational change may occur during complex formation, reorienting Arg159 and inserting the side chain into the pocket on the opposed molecule to form a salt bridge with Asp164 in *trans*. Arg159 is part of the catalytic interface (Figure 1a). Consistent with this, mutations of Arg159 had deleterious effects on oligomerisation (Additional file 12). Asp164 is not solvent exposed, but withdrawn from the surface of the protein at the bottom of a pocket. It is therefore striking that even a mild mutation like D164N prevented oligomerisation (Additional file 13). **(a and b) **View of the nucleotide-binding region. **(a) **The Irga6 dimer model (Figure 4) is shown. Arg159, Asp164 (cyan subunit) and Arg159 (magenta subunit) are shown. **(b) **A molecule of Irga6-M173A [14] is shown. Asp164 and the molecular surface formed by the residues Glu77, Thr78, Gly79, Ser80, Ile155, Ser156, Ala157, Thr158, Arg159, Phe160, Lys161, Lys162 and Asn163 are shown. **(c) **Oligomerisation of 80 μM WT or mutant Irga6 proteins was monitored by light scattering in the presence of 10 mM GTP at 37°C. **(d) **Hydrolysis of 10 mM GTP (with traces α^32^P-GTP) was measured in the presence of 80 μM WT or mutant Irga6 proteins at 37°C. Samples were assayed by TLC and autoradiography. Click here for file

Additional file 12 **Oligomerisation of Arg159 mutants**. Oligomerisation of 80 μM Irga6 mutant proteins was monitored in the presence of 10 mM GDP or GTP by DLS at 37°C. Click here for file

Additional file 13 **Oligomerisation of Asp164 mutants**. Oligomerisation of 80 μM Irga6 mutant proteins was monitored in the presence of 10 mM GDP or GTP by DLS at 20°C or 37°C. Click here for file

Additional file 14 **Binding of guanine and xanthine nucleotides to WT and Irga6-D186N**. K_d _value (μM) measured by equilibrium titration. The mean values and the standard deviation of at least two independent experiments are shown. Click here for file

Additional file 15 **The Irga6 G4-motif mutant hydrolyses GTP faster than XTP**. **(a) **Hydrolysis of 10 mM GTP (with traces α^32^P-GTP) was measured in the presence of 80 μM WT or mutant Irga6 at 37°C. Samples were assayed by TLC and autoradiography. **(b) **Hydrolysis of 10 mM XTP was measured in the presence of 80 μM WT or mutant Irga6 at 37°C. Samples were assayed by HPLC. Click here for file

Additional file 16 **The 3'OH of the GTP ribose is essential for oligomerisation; the 2'OH is not required for cooperative hydrolysis**. **(a) **Oligomerisation of 80 μM WT Irga6 protein was monitored in the presence of 10 mM GTP, 2'dGTP, 3'dGTP or 2'3'ddGTP by DLS at 37°C. **(b) **Hydrolysis of 10 mM GTP or 2'dGTP was measured after 30 min. in the presence of various concentrations of WT Irga6 protein at 37°C. Samples were assayed by HPLC. Click here for file

Additional file 17 **Oligomerisation of Glu106 mutants**. Oligomerisation of 80 μM Irga6 mutant proteins was monitored in the presence of 10 mM GDP or GTP by DLS at 37°C. Click here for file

Additional file 18 **Recruitment of Irga6 mutants to the *T. gondii *PVM**. Irga6-deficient MEFs were stimulated with IFNγ and transiently transfected with Irga6-cTag1 WT and mutant constructs. The cells were infected with avirulent *T. gondii *strain ME49. Intracellular parasites were detected with anti-GRA7 monoclonal antibody (red) and ectopically expressed Irga6-cTag1 with anti-cTag1 antiserum (green). Nuclei were stained with DAPI (blue). Irga6-cTag1 coated (arrowhead) and non-coated (arrow) parasites are indicated. Weakly coated parasites, counted as Irga6-cTag1 positive (Figure 10b), are marked with open arrowheads. Scale bar, 10 μm. Click here for file

Additional file 19 **Amino acid sequence alignment of selected G-domains**. Amino acid sequence alignment of the G-domains of Irga6, Irgb6 and Irgm3 form *Mus musculus *(MM), Ffh and FtsY from *Thermus aquaticus *(TA). The positions of G1, G3, G4 and G5 were fixed manually. Irga6 residues of the catalytic interface (Additional file 8a and 8b) are highlighted in red. Residues buried in the interface of the Ffh-FtsY dimer (PDB 1RJ9) [20] were calculated with CNSsolve [59] module buried surface [60] with a probe radius of 1.4 Å and are highlighted in red. The tendency of the interface residues to align reflects the almost equal relative spatial orientation of the G-domains in the complexes and conserved structural features of G-domains. The G1, G3, G4 and G5-motifs are highlighted by a green box. Click here for file

Additional file 20 **Conservation of the Irga6 surface**. The molecular surface of Irga6-M173A [14] is shown. ConSurf [62,63] was used with an alignment of IRGs (Additional file 2) to calculate the conservation score of Irga6 residues. Conserved residues are coloured in magenta, variable in cyan. **(a to f) **The same orientations of the molecule are shown as in Figure 1. Click here for file

Additional file 21 **Sequences of primers used for site directed mutagenesis**. List of primers (sequences 5' - 3') used for generation of the Irga6 mutants. Click here for file
