# Supplementary material for: The activation mechanism of Irga6, an interferon-inducible GTPase contributing to mouse resistance against Toxoplasma gondii
Source: BMC Biol. 2011 Jan 28;9:7. doi: 10.1186/1741-7007-9-7 (PMC3042988; doi:10.1186/1741-7007-9-7)

Additional file 1

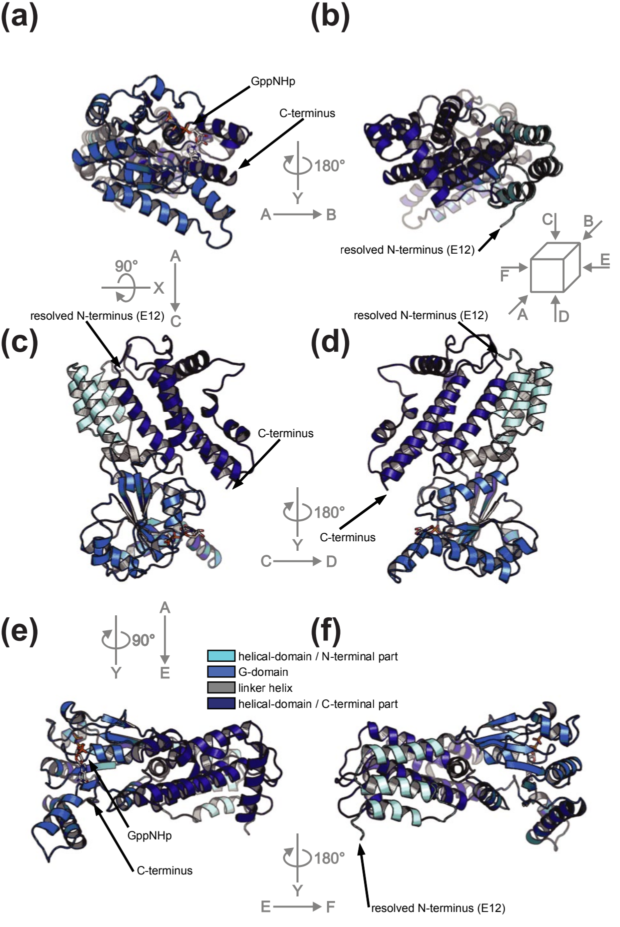

Supplement: Additional file 1 — The three-dimensional structure of Irga6. The Crystal structure of Irga6-M173A GppNHp (PDB 1TQ6) [14] is shown. Protein domains are shown as indicated in the Figure 1. (a to f) The same orientations of the molecule are shown as in Figure 1. [file 1741-7007-9-7-S1.pdf]
