# Supplementary figures and images for: The activation mechanism of Irga6, an interferon-inducible GTPase contributing to mouse resistance against Toxoplasma gondii
Source: BMC Biol. 2011 Jan 28;9:7. doi: 10.1186/1741-7007-9-7 (PMC3042988; doi:10.1186/1741-7007-9-7)

# Additional file 3

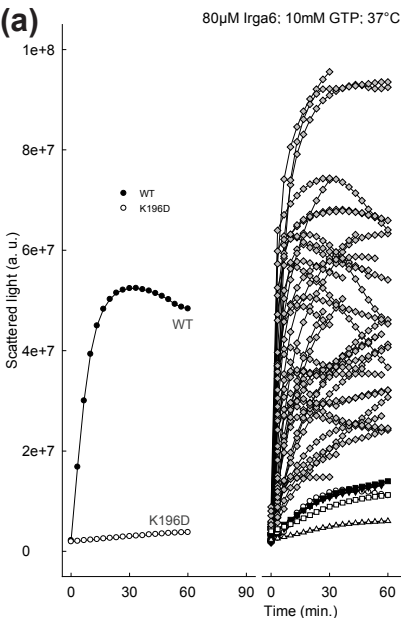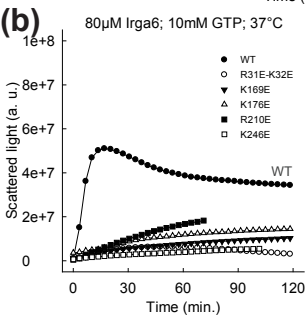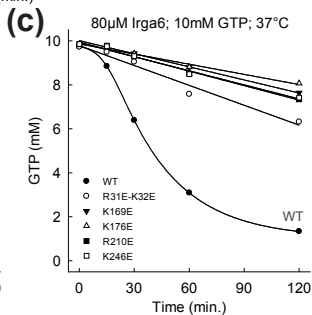

Supplement: Additional file 3 — Mutants of the secondary patch reduce oligomerisation. Mutagenesis of surface residues. (a) Oligomerisation of partially purified (see Methods) 80 μM WT or mutant Irga6 proteins was monitored by light scattering in the presence of 10 mM GTP at 37°C. Left panel: positive (WT) and negative (K196D) control (Figure 2a). Right panel: investigated mutants. Five mutants R31E-K32E, K169E, K176E, R210E and K246E inhibited the oligomerisation of Irga6, whereas many others had no significant effect. The mutants were fully purified. (b) Oligomerisation of 80 μM WT or mutant Irga6 proteins was monitored by light scattering in the presence of 10 mM GTP at 37°C. (c) Hydrolysis of 10 mM GTP (with traces α32P-GTP) was measured in the presence of 80 μM WT or mutant Irga6 proteins at 37°C. Samples were assayed by TLC and autoradiography. [file 1741-7007-9-7-S3.pdf]

# Additional file 4

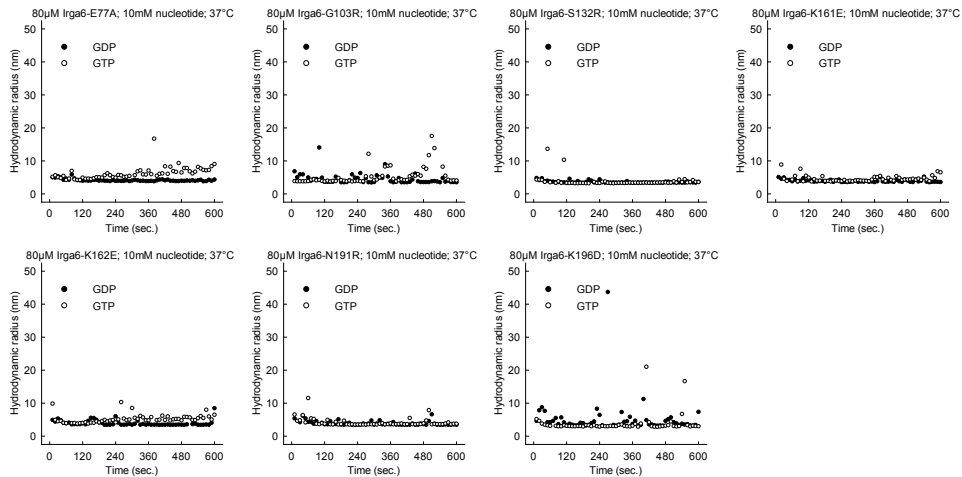

Supplement: Additional file 4 — Oligomerisation of the catalytic interface mutants. Oligomerisation of 80 μM Irga6 mutant proteins was monitored in the presence of 10 mM GDP or GTP by DLS at 37°C. [file 1741-7007-9-7-S4.pdf]

# Additional file 7

(a)

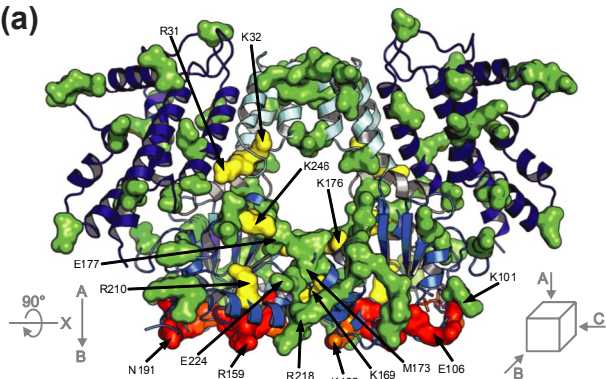

(b)

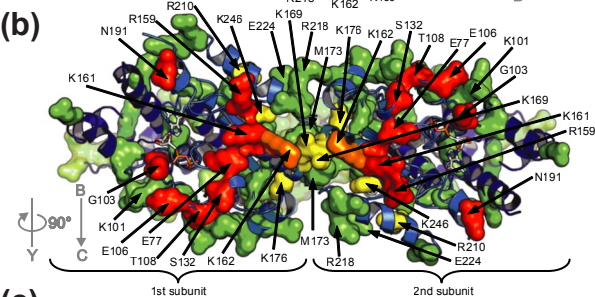

(c)

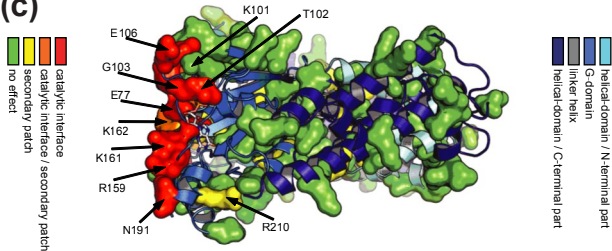

Supplement: Additional file 7 — Position of mutated residues in the crystal dimer. The Irga6 crystal dimer (PDB 1TPZ) [14] is shown. Protein domains and mutated residues are shown as indicated in the Figure 1. Lys9, Ser10, Lys196 of both subunits and Lys202 of the second subunit are not resolved in the crystal structure. (a) Top view. (b) Front view of the two G-domains; Additional file 7a rotated by 90° around the x-axis. (c) Left view; Additional file 7b rotated by 90° around the y-axis. [file 1741-7007-9-7-S7.pdf]

# Additional file 8

(a)

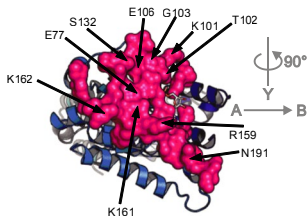

(b)

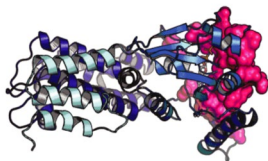

(c)

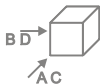

(d)

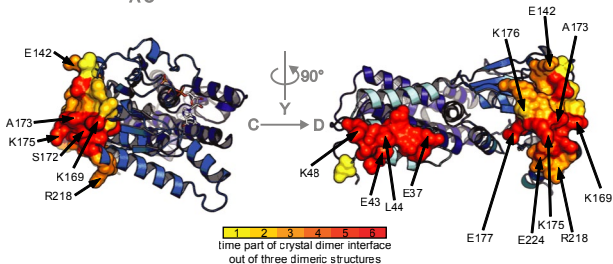

Supplement: Additional file 8 — Relative position of catalytic and crystal dimer interface. The structure of Irga6-M173A [14] is shown. Protein domains are shown as indicated in the Figure 1. (a and b) Residues buried in the interface of the Irga6 dimer model were calculated with CNSsolve [59] module buried surface [60] with a probe radius of 1.4 Å. The surface formed by Glu77, Thr78, Gly79, Asn94, Glu95, Lys101, Thr102, Gly103, Glu106, Val107, Gly131, Ser132, Thr133, Pro136, Pro137, Ala157, Thr158, Arg159, Phe160, Lys161, Lys162, Asn163, Asp166, Lys184, Asp186, Ser187, Asp188, Thr190, Asn191, Asp194, Gly195 and Lys233 is shown in magenta. (c and d) Residues buried in the crystal dimer interface were calculated by the same method. The two surfaces formed by Asn14, Ser18, Gln36, Glu37, Asn40, Leu41, Glu43, Leu44, Arg47, Lys48, Pro137, Asn138, Thr139, Leu141, Glu142, Tyr147, Asp166, Ala168, Lys169, Ala170, Ser172, Ala173 (instead of Met173), Met174, Lys175, Lys176, Glu177, Phe178, Arg218, Gly221, Ile222, Ala223 and Glu224 are shown. Three dimeric crystal structures of Irga6 are available (PDB 1TPZ, 1TQ2 and 1TQD) [14] therefore each residue can be maximum six time involved in this interface. Residues highly relevant for the crystal dimer interface are shown in red, less relevant in yellow. (a and c) Front view of the G-domain (Figure 1a). (b and d) Left view (Figure 1f). [file 1741-7007-9-7-S8.pdf]

# Additional file 9

(a)

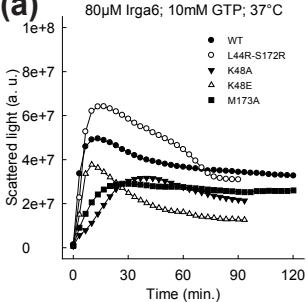

(b)

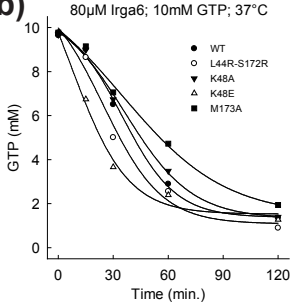

Supplement: Additional file 9 — Mutations of the crystal dimer interface do not prevent oligomerisation. (a) Oligomerisation of 80 μM WT or mutant Irga6 proteins was monitored by light scattering in the presence of 10 mM GTP at 37°C. (b) Hydrolysis of 10 mM GTP (with traces α32P-GTP) was measured in the presence of 80 μM WT or mutant Irga6 proteins at 37°C. Samples were assayed by TLC and autoradiography. [file 1741-7007-9-7-S9.pdf]

# Additional file 11

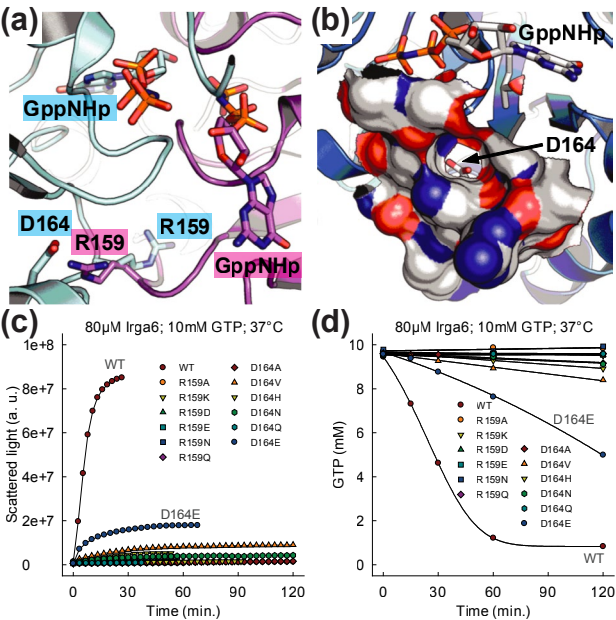

Supplement: Additional file 11 — Asp164 and Arg159 participate in oligomerisation. For the construction of the Irga6 dimer model a rigid crystal structure was used. In the model the side chains of the Arg159 residues of the two subunits collide. Arg159 is located close to Asp164 on the other subunit. Asp164 forms the bottom of a pocket, derived from two loops. One loop is located between Glu77 and Ser80 and contains a part of the G1-motif. The other loop is located between Ile155 and Asn163. The conformation of Arg159 is relatively unconstrained [14]. A conformational change may occur during complex formation, reorienting Arg159 and inserting the side chain into the pocket on the opposed molecule to form a salt bridge with Asp164 in trans. Arg159 is part of the catalytic interface (Figure 1a). Consistent with this, mutations of Arg159 had deleterious effects on oligomerisation (Additional file 12). Asp164 is not solvent exposed, but withdrawn from the surface of the protein at the bottom of a pocket. It is therefore striking that even a mild mutation like D164N prevented oligomerisation (Additional file 13). (a and b) View of the nucleotide-binding region. (a) The Irga6 dimer model (Figure 4) is shown. Arg159, Asp164 (cyan subunit) and Arg159 (magenta subunit) are shown. (b) A molecule of Irga6-M173A [14] is shown. Asp164 and the molecular surface formed by the residues Glu77, Thr78, Gly79, Ser80, Ile155, Ser156, Ala157, Thr158, Arg159, Phe160, Lys161, Lys162 and Asn163 are shown. (c) Oligomerisation of 80 μM WT or mutant Irga6 proteins was monitored by light scattering in the presence of 10 mM GTP at 37°C. (d) Hydrolysis of 10 mM GTP (with traces α32P-GTP) was measured in the presence of 80 μM WT or mutant Irga6 proteins at 37°C. Samples were assayed by TLC and autoradiography. [file 1741-7007-9-7-S11.pdf]

# Additional file 12

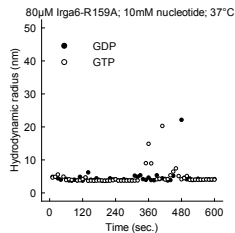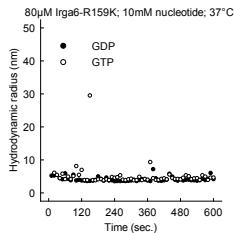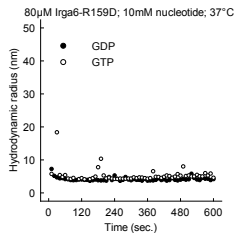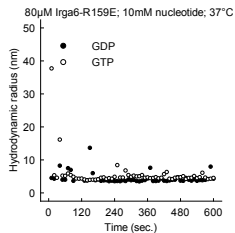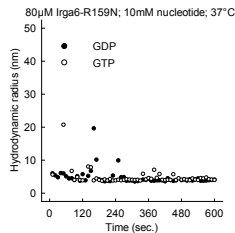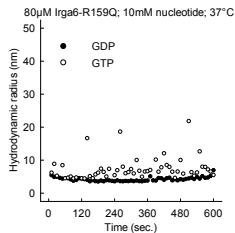

Supplement: Additional file 12 — Oligomerisation of Arg159 mutants. Oligomerisation of 80 μM Irga6 mutant proteins was monitored in the presence of 10 mM GDP or GTP by DLS at 37°C. [file 1741-7007-9-7-S12.pdf]

# Additional file 13

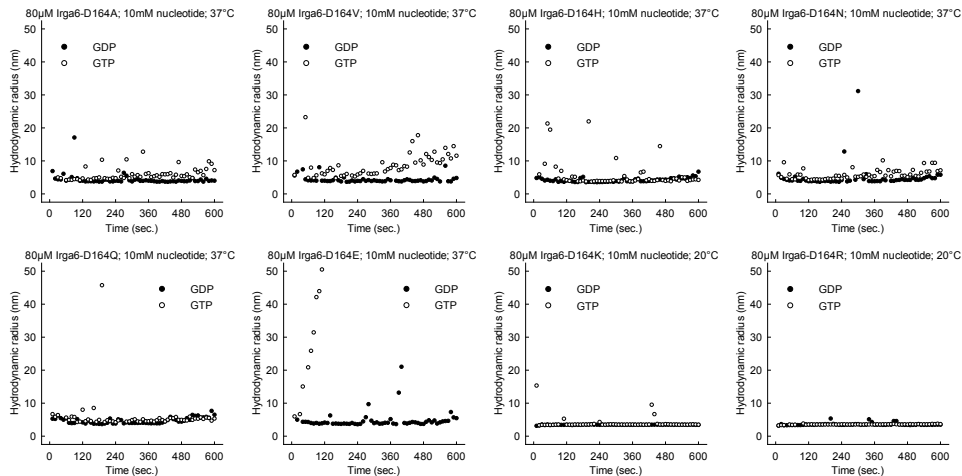

Supplement: Additional file 13 — Oligomerisation of Asp164 mutants. Oligomerisation of 80 μM Irga6 mutant proteins was monitored in the presence of 10 mM GDP or GTP by DLS at 20°C or 37°C. [file 1741-7007-9-7-S13.pdf]

# Additional file 15

(a)

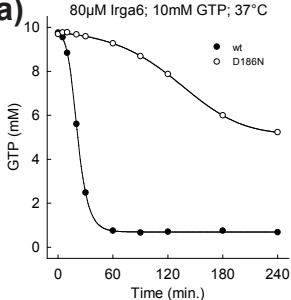

(b)

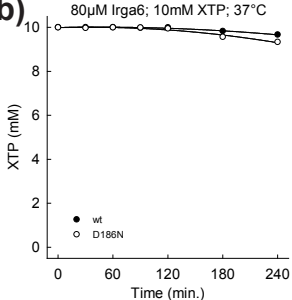

Supplement: Additional file 15 — The Irga6 G4-motif mutant hydrolyses GTP faster than XTP. (a) Hydrolysis of 10 mM GTP (with traces α32P-GTP) was measured in the presence of 80 μM WT or mutant Irga6 at 37°C. Samples were assayed by TLC and autoradiography. (b) Hydrolysis of 10 mM XTP was measured in the presence of 80 μM WT or mutant Irga6 at 37°C. Samples were assayed by HPLC. [file 1741-7007-9-7-S15.pdf]

# Additional file 16

(a)

80  $\mu$ M Irga6; 10mM nucleotide; 37°C

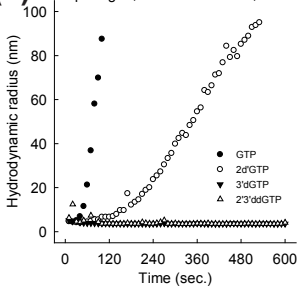

(b)

10mM nucleotide; 37°C; 30min.

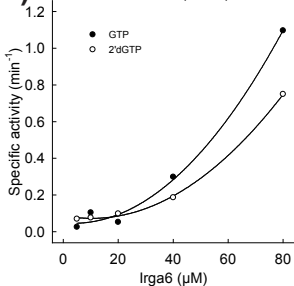

Supplement: Additional file 16 — The 3'OH of the GTP ribose is essential for oligomerisation; the 2'OH is not required for cooperative hydrolysis. (a) Oligomerisation of 80 μM WT Irga6 protein was monitored in the presence of 10 mM GTP, 2'dGTP, 3'dGTP or 2'3'ddGTP by DLS at 37°C. (b) Hydrolysis of 10 mM GTP or 2'dGTP was measured after 30 min. in the presence of various concentrations of WT Irga6 protein at 37°C. Samples were assayed by HPLC. [file 1741-7007-9-7-S16.pdf]

# Additional file 17

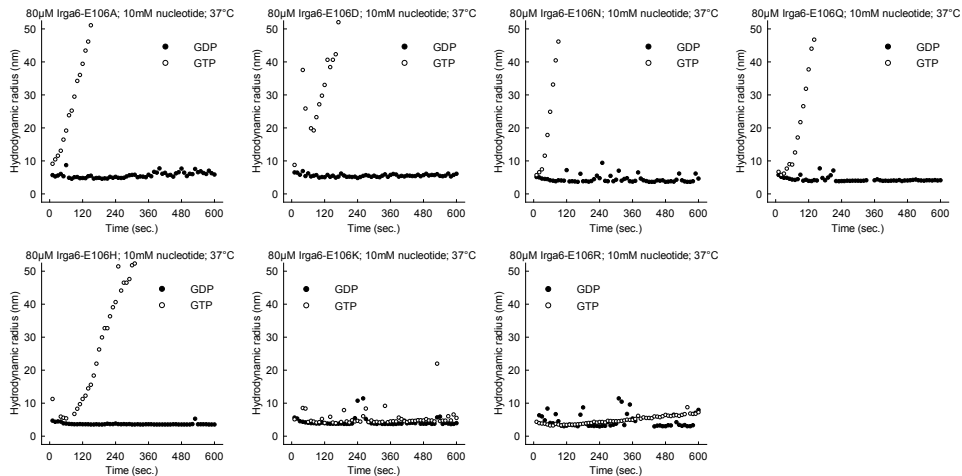

Supplement: Additional file 17 — Oligomerisation of Glu106 mutants. Oligomerisation of 80 μM Irga6 mutant proteins was monitored in the presence of 10 mM GDP or GTP by DLS at 37°C. [file 1741-7007-9-7-S17.pdf]

# Additional file 18

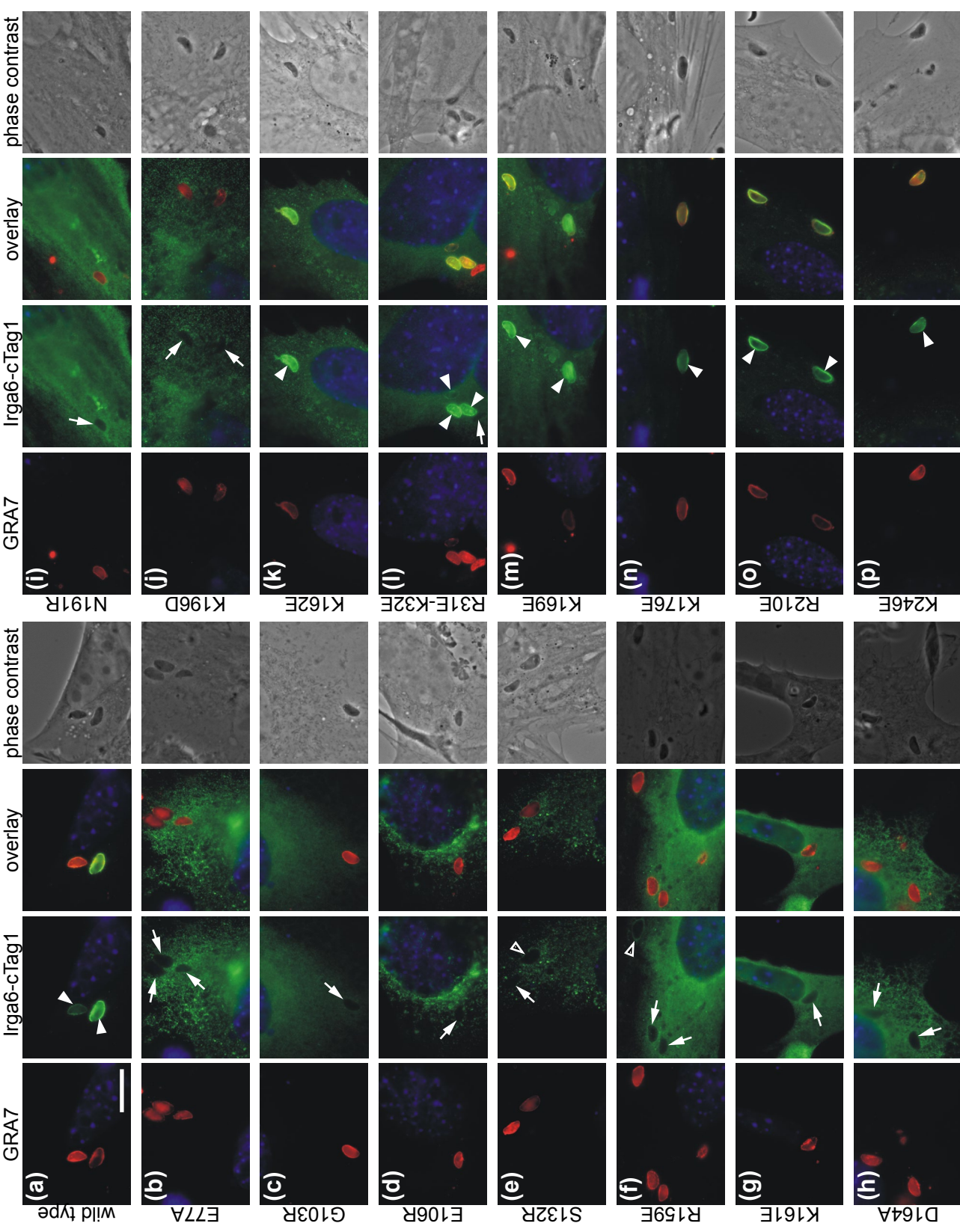

Supplement: Additional file 18 — Recruitment of Irga6 mutants to the T. gondii PVM. Irga6-deficient MEFs were stimulated with IFNγ and transiently transfected with Irga6-cTag1 WT and mutant constructs. The cells were infected with avirulent T. gondii strain ME49. Intracellular parasites were detected with anti-GRA7 monoclonal antibody (red) and ectopically expressed Irga6-cTag1 with anti-cTag1 antiserum (green). Nuclei were stained with DAPI (blue). Irga6-cTag1 coated (arrowhead) and non-coated (arrow) parasites are indicated. Weakly coated parasites, counted as Irga6-cTag1 positive (Figure 10b), are marked with open arrowheads. Scale bar, 10 μm. [file 1741-7007-9-7-S18.pdf]

# Additional file 20

(a)

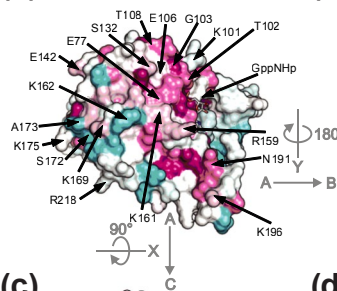

(b)

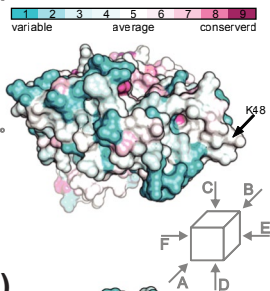

(c)

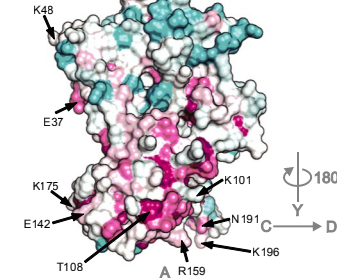

(d)

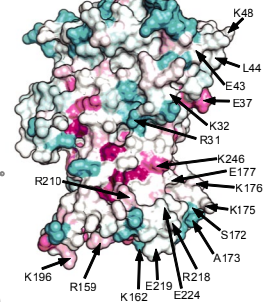

(e)

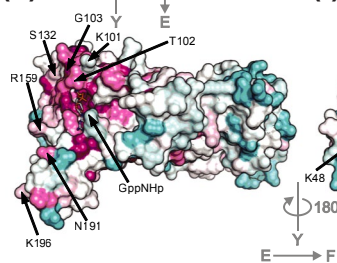

(f)

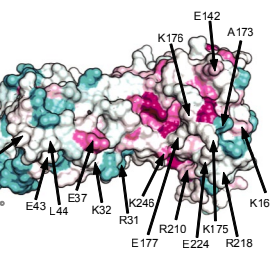

Supplement: Additional file 20 — Conservation of the Irga6 surface. The molecular surface of Irga6-M173A [14] is shown. ConSurf [62,63] was used with an alignment of IRGs (Additional file 2) to calculate the conservation score of Irga6 residues. Conserved residues are coloured in magenta, variable in cyan. (a to f) The same orientations of the molecule are shown as in Figure 1. [file 1741-7007-9-7-S20.pdf]
