# Supplementary material for: The activation mechanism of Irga6, an interferon-inducible GTPase contributing to mouse resistance against Toxoplasma gondii
Source: BMC Biol. 2011 Jan 28;9:7. doi: 10.1186/1741-7007-9-7 (PMC3042988; doi:10.1186/1741-7007-9-7)

Additional file 5

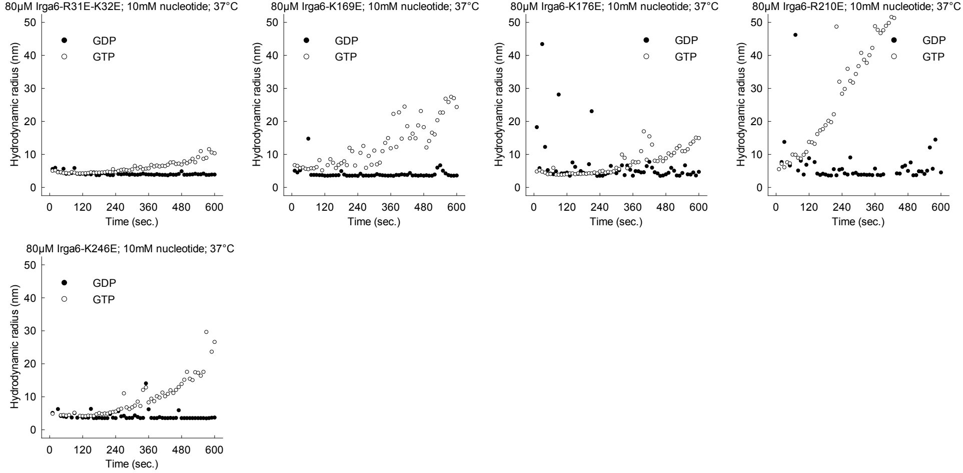

Supplement: Additional file 5 — Oligomerisation of the secondary patch mutants. Oligomerisation of 80 μM Irga6 mutant proteins was monitored in the presence of 10 mM GDP or GTP by DLS at 37°C. [file 1741-7007-9-7-S5.pdf]
