# Supplementary material for: The activation mechanism of Irga6, an interferon-inducible GTPase contributing to mouse resistance against Toxoplasma gondii
Source: BMC Biol. 2011 Jan 28;9:7. doi: 10.1186/1741-7007-9-7 (PMC3042988; doi:10.1186/1741-7007-9-7)
Supplement: Additional file 6 — Nucleotide-binding affinities of oligomerisation impaired Irga6 mutants. Dissociation constant (Kd) measured by equilibrium titration. The mean values and the standard deviation of at least two independent experiments are shown. [file 1741-7007-9-7-S6.pdf]

Additional file 6

Irga6	nucleotide	Kd (µM)
WT	mant-GTPγS	18.3 ± 5.1
E77A	mant-GTPγS	33.9 ± 5.9
G103R	mant-GTPγS	21.3 ± 9.4
E106R	mant-GTPγS	13.2 ± 4.6
S132R	mant-GTPγS	20 ± 10.6
R159E	mant-GTPγS	41.2 ± 12.7
K161E	mant-GTPγS	49.5 ± 20.8
D164A	mant-GTPγS	24.8 ± 4.7
N191R	mant-GTPγS	34.1 ± 11
K196D	mant-GTPγS	26.8 ± 9.7
K162E	mant-GTPγS	22.9 ± 6.9
R31E-K32E	mant-GTPγS	54.5 ± 7.7
K169E	mant-GTPγS	47.9 ± 5.8
K176E	mant-GTPγS	112.8 ± 31
R210E	mant-GTPγS	158.5 ± 61.1
K246E	mant-GTPγS	48.2 ± 4
