# Supplementary material for: The activation mechanism of Irga6, an interferon-inducible GTPase contributing to mouse resistance against Toxoplasma gondii
Source: BMC Biol. 2011 Jan 28;9:7. doi: 10.1186/1741-7007-9-7 (PMC3042988; doi:10.1186/1741-7007-9-7)
Supplement: Additional file 14 — Binding of guanine and xanthine nucleotides to WT and Irga6-D186N. Kd value (μM) measured by equilibrium titration. The mean values and the standard deviation of at least two independent experiments are shown. [file 1741-7007-9-7-S14.pdf]

Additional file 14

nucleotide	WT	D186N
mant-GTP	26.6 ± 5	96.9 ± 16
2'deoxy-3'mant-GTP	38.3 ± 7.7	
2'mant-3'deoxy-GTP	58.4 ± 11.6	
mant-GDP	1.4 ± 0.1	62.6 ± 28.3
mant-XTP	58.5 ± 8.1	11.2 ± 0.8
mant-XDP	10 ± 1.2	0.8 ± 0.3
