# Supplementary material for: The activation mechanism of Irga6, an interferon-inducible GTPase contributing to mouse resistance against Toxoplasma gondii
Source: BMC Biol. 2011 Jan 28;9:7. doi: 10.1186/1741-7007-9-7 (PMC3042988; doi:10.1186/1741-7007-9-7)
Supplement: Additional file 19 — Amino acid sequence alignment of selected G-domains. Amino acid sequence alignment of the G-domains of Irga6, Irgb6 and Irgm3 form Mus musculus (MM), Ffh and FtsY from Thermus aquaticus (TA). The positions of G1, G3, G4 and G5 were fixed manually. Irga6 residues of the catalytic interface (Additional file 8a and 8b) are highlighted in red. Residues buried in the interface of the Ffh-FtsY dimer (PDB 1RJ9) [20] were calculated with CNSsolve [59] module buried surface [60] with a probe radius of 1.4 Å and are highlighted in red. The tendency of the interface residues to align reflects the almost equal relative spatial orientation of the G-domains in the complexes and conserved structural features of G-domains. The G1, G3, G4 and G5-motifs are highlighted by a green box. [file 1741-7007-9-7-S19.pdf]

Additional file 19

```
MM_Irga6   SVLNVAVTGETGSGKSSFIN-------------------TLRGIGNEEE----GAAKTGV
MM_Irgb6   APLHIAVTGETGAGKSTFIN-------------------TLRGVGHEEK----GAAPTGA
MM_Irgm3   YRVKIAVTGDSGNGMSSFIN-------------------ALRFIGHEEE----DSAPTGV
TA_Ffh     -RNLWFLVGLQGSGKTTTAAKLALYYKGKGRRPLLVAADTQRPAAREQLRLLGEKVGVPV
TA_FtsY    -GRVVLVVGVNGVGKTTTIAKLGRYYQNLGKKVMFCAGDTFRAAGGTQLSEWGKRLSIPV
```

G1

```
MM_Irga6   VEVT--------MERHPYK-HPNIPNVVFWDLPGIGSTNFPPNTYLEKMKFYEY------
MM_Irgb6   IETT--------MKRTPYP-HPKLPNVTIWDLPGIGTTNFTPQNYLTEMKFGEY------
MM_Irgm3   VRTT--------KKPACYSSDSHFPYVELWDLPGLGATAQSVESYLEEMQISTF------
TA_Ffh     LEVMDGESPESIRRVEEKARLEARDLILVDTAGRLQIDEPLMGELARLKEVLG------
TA_FtsY    IQGPEGTDSAALAYDAVQAMKARGYDLLFVDTAGRLHTKHNLMEELKKVKRAIAKADPEE
```

G3

```
MM_Irga6   -DFFIIISATRFKKNDIDIAKAISMMKKEFYFVRTKVDSDITNEADGKPQTFDKEKVLQD
MM_Irgb6   -DFFIIISATRFKENDAQLAKAIAQMGMNFYFVRTKIDSDLDNEQKFKPKSFNKEEVLKN
MM_Irgm3   -DLIIIVASEQFSSNHVKLAITMQRMRKRFYVVWTKLDRDLSTS------TFPEPQLLQS
TA_Ffh     PDEVLLVLDAMTGQEALSVARAFDEKVGVTGLVLTKLDGDARGG------------AALS
TA_FtsY    PKEVWLVLDAVTGQNGLEQAKKFHEAVGLTGVIVTKLDGTAKGG------------VLIP
```

G4

```
MM_Irga6   IRLNCVNTFRENGIAEPPIFLLSNK--NVCHYDFPVLMDKLISDLP----
MM_Irgb6   IKDYCSNHLQESLDSEPPVFLVSNV--DISKYDFPKLETKLLQDLP----
MM_Irgm3   IQRNIRENLQQAQVRDPPLFLISCF--SPSFHDFPELRNTLQKDIF----
TA_Ffh     ARHVTGKPIYFAG--------VSEKPEGLEPFYPERLAGRILGMGDVASL
TA_FtsY    IVRTLKVPIKFVG--------VGEGPDDLQPFDPEAFVEALLED------
```

G5
