# Supplementary material for: The activation mechanism of Irga6, an interferon-inducible GTPase contributing to mouse resistance against Toxoplasma gondii
Source: BMC Biol. 2011 Jan 28;9:7. doi: 10.1186/1741-7007-9-7 (PMC3042988; doi:10.1186/1741-7007-9-7)
Supplement: Additional file 21 — Sequences of primers used for site directed mutagenesis. List of primers (sequences 5' - 3') used for generation of the Irga6 mutants. [file 1741-7007-9-7-S21.pdf]

Additional file 21

mutation	primer sequence 5' - 3'
K9E-S10R	gggtcagctgttctcttcacctgagcgtgatgagaataatgatttgccc
S18R	gaataatgatttgccccgcagctttactgg
R31E-K32E	ggttattttaagaaatttaatacgggagaagaaatcatttctcaagagatcctcaatttg
E37R	gaaaaatcatttctcaaaggatcctcaatttg
E43R	caagagatcctcaatttgattagattaaggatgagaaaagggaatattc
N50R	ggatgagaaaagggaggattcagttgacaaac
Q52R	gaaaagggaatattcgcttgacaaactctgc
S56R	cagttgacaaaccgtgcaatcagtgatgc
E64K	gtgatgcattaaaaaaaatcgatagtagtgtgc
E77A	gctcaatgttgctgtcaccggggcgacgggatcagggaagtcc
T88R	ggatcagggaagtccagcttcatcaatcgcctgagaggcattgggaatgaagaagaagg
E97R	gggaatgaagaacgaggtgcagctaaaactggg
K101E	gaagaagaaggtgcagctgaaactggggtggtggaggtaacc
G103R	ggaatgaagaagaaggtgcagctaaaactagggtggtggaggtaaccatggaaagacatc
E106A	gctaaaactggggtggtggcggtaaccatggaaag
E106D	ggtgcagctaaaactggggtggtggacgtaaccatggaaagacatcc
E106N	ggtgcagctaaaactggggtggtgaacgtaaccatggaaagacatcc
E106Q	ggtgcagctaaaactggggtggtgcaggtaaccatggaaagacatcc
E106H	ggtgcagctaaaactggggtggtgcacgtaaccatggaaagacatcc
E106K	gctaaaactggggtggtgaaggtaaccatggaaag
E106R	gctaaaactggggtggtgcgggtaaccatggaaag
M109A	ggtggaggtaaccgcggaaagacatccatac
E110R-R111E	ctggggtggtggaggtaaccatgagagaacatccatacaaacaccccaatataccc
K115E	ggtaaccatggaaagacatccatacgaacaccccaatatacccaatgtgg
S132R	gggacctgcctgggattggaaggacaaatttcccaccaaac
E142R	ccaaacacttacctgcggaaaatgaagttctatg
K145E	cctggagaaaatggagttctatgagtacgatttc
E148R	gaaaatgaagttctatcggtacgatttcttcattattatttcg
D150R	ggagaaaatgaagttctatgagtaccgtttcttcattattatttcggccacacgc
R159A	cgatttcttcattattatttcggccacagccttcaagaaaaatgatatagacattgcc
R159K	cgatttcttcattattatttcggccacaaagttcaagaaaaatgatatagacattgcc
R159D	cgatttcttcattattatttcggccacagacttcaagaaaaatgatatagacattgcc
R159E	cattattatttcggccacagaattcaagaaaaatgatatag
R159N	cgatttcttcattattatttcggccacaaacttcaagaaaaatgatatagacattgcc
R159Q	cgatttcttcattattatttcggccacacaattcaagaaaaatgatatagacattg
K161E	cggccacacgcttcgagaaaaatgatatagac
K162E	gccacacgcttcaaggaaaatgatatagacattgc
D164A	cggccacacgcttcaagaaaaatgctatagacattgccaaagcaatcagc
D164V	cggccacacgcttcaagaaaaatgttatagacattgccaaagcaatcagc
D164H	cggccacacgcttcaagaaaaatcatatagacattgccaaagcaatcagc

mutation	primer sequence 5' - 3'
D164N	cggccacacgcttcaagaaaaataatatagacattgccaaagcaatcagc
D164Q	cggccacacgcttcaagaaaaatcaaatagacattgccaaagcaatcagc
D164E	cggccacacgcttcaagaaaaatgagatagacattgccaaagcaatcagc
D164K	cggccacacgcttcaagaaaaataaaatagacattgccaaagcaatcagc
D164R	cggccacacgcttcaagaaaaatcgtatagacattgccaaagcaatcagc
K169E	gaaaaatgatatagacattgccgaagcaatcagcatgatg
M173E	cattgccaaagcaatcagcgagatgaagaaggaattctac
M173R	cattgccaaagcaatcagcaggatgaagaaggaattctac
M173W	cattgccaaagcaatcagctggatgaagaaggaattctac
K175E	gcaatcagcatgatggagaaggaattctacttcg
K176E	gcaatcagcatgatgaaggaggaattctacttcgtg
E177R	caaagcaatcagcatgatgaagaagcgattctacttcgtgagaaccaaggtgg
D186N	gtgagaaccaaggtgaattctgacataacaaatg
N191R	ggtggactctgacataacaagagaagcagatggcaaacctc
K196D	gaagcagatggcgaccctcaaacctttgac
K202A	cctcaaacctttgacgcagaaaaggtcctgc
E203R	cctttgacaaaagaaaggtcctgcagg
R210E	gacaaagaaaaggtcctgcaggacatcgagcttaactgtgtgaacacctttaggg
R218E-E219R	ccgccttaactgtgtgaacacctttgagaggaatggcattgctgagccaccaatc
E224R	cacctttagggagaatggcattgctcggccaccaatcttcctgctctctaac
H237D	caaaaatgtttgtaactatgacttccccg
V242R	gtttgtcactatgacttcccccgcctgatggacaagctgataagtgacc
D245R	gtcactatgacttccccgtcctgatgcgcaagctgataagtgacctccctatc
K246E	ctatgacttccccgtcctgatggacgagctgataagtgacctccctatctac
D250R	cccgtcctgatggacaagctgataagtcgcctccctatctacaagagacac
K255E	gctgataagtgacctccctatctacgagagacacaattttatggtctccttaccc
N265R	gagacacaattttatggtctccttaccccgtatcacagattcagtcattgaaaagaagc
S269R	ggtctccttacccaatatcacagatcgagtcattgaaaagaagcggcaatttc
R275E	cagattcagtcattgaaaagaaggagcaatttctgaagcagaggatttggc
E285R	gcggcaatttctgaagcagaggatttggctgcgaggatttgctgctgacctagtg
N293R	ggaaggatttgctgctgacctagtgcgtatcatcccttctctgacctttctcttgg
S304R	cccttctctgacctttctcttggaccgtgatttggagactctgaagaaaagcatg
K310E-K311E	cttggacagtgatttggagactctggaggaaagcatgaaattctaccgcactgtg
T325R-S326R	ccgcactgtgtttggagtggatgaaagacgtttgcagagattagctagggactggg
E335R	ctttgcagagattagctagggactggcgaatagaggtggatcaggtggaggcc
K346E	ggtggatcaggtggaggccatgatagaatctcctgctgtgttcaaacctacag
D355R-E356R-E357R	aatctcctgctgtgttcaaacctacacgtcgacgaacaatacaagaaaggctttcaagat
L372R-A373R	gctttcaagatatattcaggagttctgtcggcgtaatgggtacttacttcctaaaaatag
K407E	ggtgactgaggatgctaaaactcttcttgaagagatatgtttaagaaactag
