# Supplementary material for: The activation mechanism of Irga6, an interferon-inducible GTPase contributing to mouse resistance against Toxoplasma gondii
Source: BMC Biol. 2011 Jan 28;9:7. doi: 10.1186/1741-7007-9-7 (PMC3042988; doi:10.1186/1741-7007-9-7)

Additional file 2

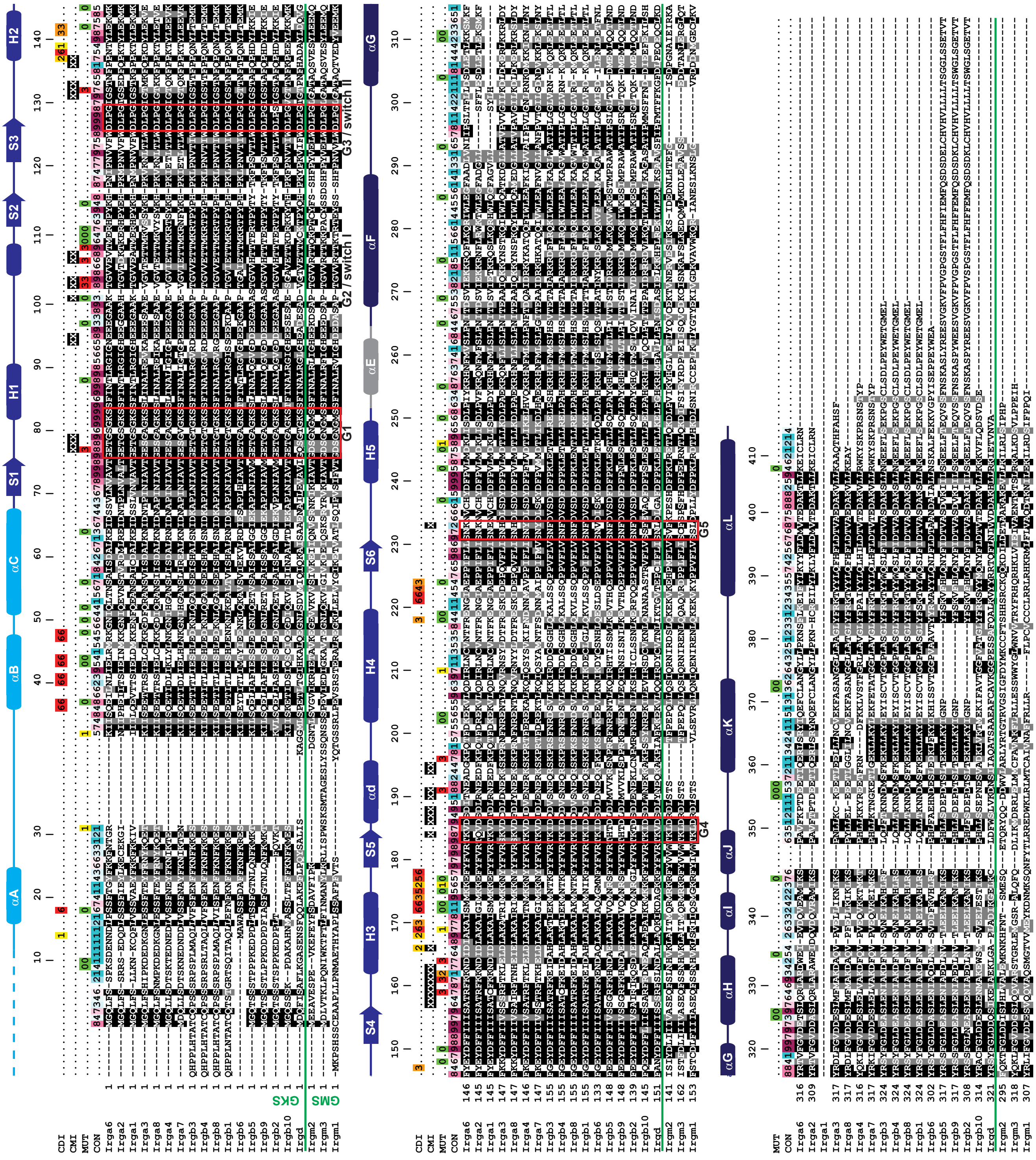

Supplement: Additional file 2 — Amino acid sequence alignment of mouse IRGs. Amino acid sequence alignment of Irga1, Irga2, Irga3, Irga4, Irga6, Irga7, Irga8, Irgb1, Irgb2, Irgb3, Irgb4, Irgb5, Irgb6, Irgb8, Irgb9, Irgb10, Irgd, Irgm1, Irgm2 and Irgm3 from the C57BL/6 mouse. Irgc is not induced by IFNγ, Irga5 and Irgb7 are pseudogenes [4] and were thus excluded. Residues relevant for the crystal dimer interface (CDI) (Additional file 8c and 8d) are highlighted (yellow 1 - red 6; indication how often they form part of the crystal dimer interface in the three available dimeric structures of Irga6 [14]). Residues that are part of the catalytic interface (CI) (Additional file 8a, b) are marked (black X). Residues mutagenised (MUT) in this study and by Steinfeldt et al. [22] (Figure 1) are indicated: no inhibition of oligomerisation (green 0), inhibition of oligomerisation and part of the secondary patch (yellow 1), inhibition of oligomerisation and part of the secondary patch or the catalytic interface (orange 2), inhibition of oligomerisation and part of the catalytic interface (red 3). The calculated conservation score (CON) (Additional file 20) is displayed: variable (cyan 1) - conserved (magenta 9). The G1, G3, G4 and G5-motifs are highlighted by a red box. The GKS and GMS subfamilies are separated by a green line. [file 1741-7007-9-7-S2.pdf]
